# Combining Crystallization-Driven Self-Assembly with Reverse Sequence Polymerization-Induced Self-Assembly Enables the Efficient Synthesis of Hydrolytically Degradable Anisotropic Block Copolymer Nano-objects Directly in Concentrated Aqueous Media

**DOI:** 10.1021/jacs.4c06299

**Published:** 2024-06-06

**Authors:** Matthew A. H. Farmer, Osama M. Musa, Steven P. Armes

**Affiliations:** † Department of Chemistry, University of Sheffield, Dainton Building, Brook Hill, Sheffield, South Yorkshire S3 7HF, U.K.; ‡ Ashland Specialty Ingredients, 1005 US 202/206, Bridgewater, New Jersey 08807, United States

## Abstract

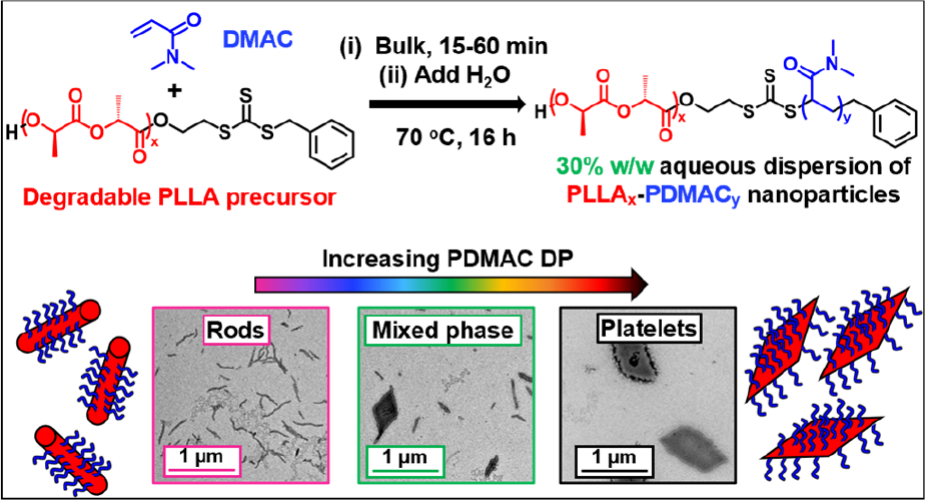

Herein we combine the well-known processing advantages conferred by polymerization-induced self-assembly (PISA) with crystallization-driven self-assembly (CDSA) to achieve the efficient synthesis of hydrolytically degradable, highly anisotropic block copolymer nano-objects directly in aqueous solution at 30% w/w solids. This new strategy involves a so-called reverse sequence PISA protocol that employs poly(l-lactide) (PLLA) as the crystallizable core-forming block and poly(*N,N′*-dimethylacrylamide) (PDMAC) as the water-soluble non-ionic coronal block. Such syntheses result in PDMAC-rich anisotropic nanoparticles. Depending on the target diblock copolymer composition, either rod-like nanoparticles or diamond-like platelets can be obtained. Furthermore, *N*-Acryloylmorpholine is briefly evaluated as an alternative hydrophilic vinyl monomer to DMAC. Given that the PLLA block can undergo either hydrolytic or enzymatic degradation, such nanoparticles are expected to offer potential applications in various fields, including next-generation sustainable Pickering emulsifiers.

## Introduction

Following pioneering studies by Manners and Winnik, crystallization-driven self-assembly (CDSA) is now well-established as a synthetic route to highly anisotropic block copolymer rods and other interesting morphologies such as diamond-like platelets.^1−11^ CDSA typically utilizes an insoluble crystalline block and a soluble steric stabilizer block. Most studies are conducted at high dilution in a range of organic solvents, but CDSA can also be achieved in (dilute) aqueous solution, which is preferred for potential bioapplications.^7,12−15^

**Scheme 1:**
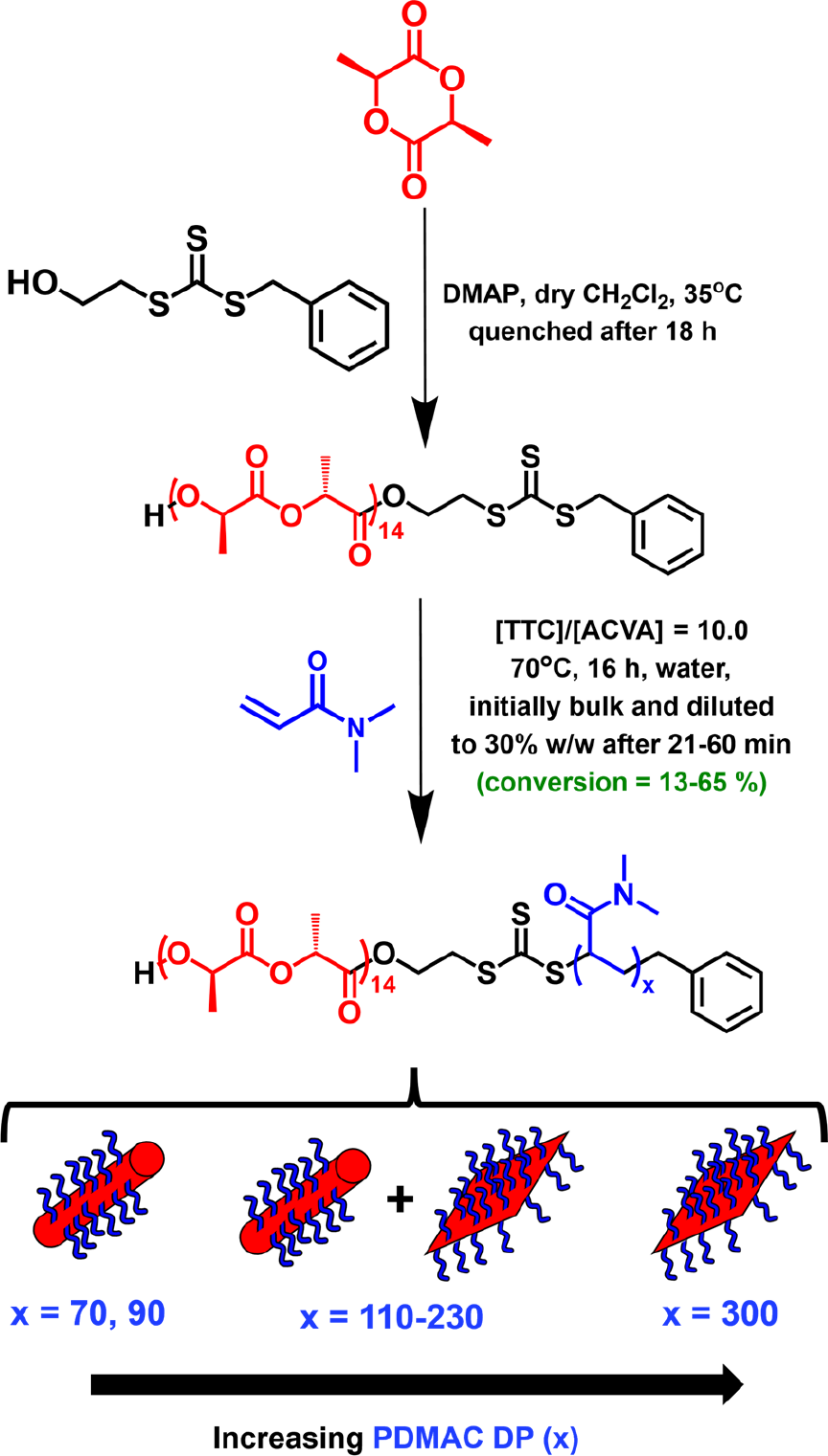
Synthesis of a 30% w/w Aqueous Dispersion of PLLA_14_-PDMAC_x_ Diblock Copolymer Nanoparticles by the Judicious Combination of Reverse Sequence PISA with CDSA A PLLA_14_-TTC precursor is first prepared via anionic ring-opening polymerization of l-lactide at 35 °C using a hydroxy-functional RAFT agent as an initiator. The RAFT polymerization of DMAC is then conducted in the bulk at 70 °C. At a suitable intermediate DMAC conversion, the reaction mixture is diluted with deionized water to induce self-assembly of the growing amphiphilic PLLA_14_-PDMAC_x_ diblock copolymer chains.

Unlike CDSA, polymerization-induced self-assembly (PISA) normally involves growing an insoluble amorphous block from a soluble precursor block.^16−21^ Depending on the target diblock copolymer composition, this approach typically yields spheres, worms, or vesicles.^22^ Recently, we reported a counterintuitive reverse sequence aqueous PISA formulation in which a hydrophobic precursor is solubilized in concentrated aqueous media using a water-miscible vinyl monomer as a cosolvent.^23^ Polymerization of this monomer gradually worsens the solvency for the growing amphiphilic diblock copolymer chains, which subsequently undergo *insitu* self-assembly to form spheres. Herein, we combine the processing advantages offered by reverse sequence PISA with CDSA to prepare highly anisotropic hydrolytically degradable block copolymer nano-objects directly in aqueous media at 30% w/w solids.^24^ This new strategy involves a hydroxy-functional trithiocarbonate reagent,^7,10,25,26^ employs poly(l-lactide) (PLLA) as the crystallizable core-forming block, and uses poly(*N,N′*-dimethylacrylamide) (PDMAC) as the water-soluble non-ionic coronal block (see Scheme 1).

## Results and Discussion

Recently, we reported a reverse sequence PISA formulation based on a hydrophobic poly(ε-caprolactone) (PCL) precursor, which exhibits a melting transition, *T*_m_, at approximately 50 °C. In this prior study, the *in situ* DMAC polymerization was performed at 80 °C, which results in the formation of spherical PCL–PDMAC nanoparticles with amorphous cores.^23^ In contrast, PLLA has a *T*_m_ of 114–153 °C (see Figure S1). Hence reverse sequence PISA syntheses performed at 70 °C should lead to the formation of anisotropic PLLA-PDMAC nanoparticles with semicrystalline cores via CDSA (see Scheme 1).

In the present study, the anionic ring-opening polymerization of l-lactide was initiated using a hydroxy-functional reversible addition–fragmentation chain transfer (RAFT) agent in the presence of a 4-(dimethylamino)pyridine (DMAP) catalyst, as previously reported.^25^ Anhydrous conditions ensured controlled polymerization to produce a hydrophobic semicrystalline poly(l-lactide) PLLA precursor with a mean degree of polymerization (DP) of 14 as determined by end-group analysis using ^1^H NMR spectroscopy. More specifically, the integrated oxymethine PLLA signal at 5.21 ppm was compared to the integrated aromatic proton signals assigned to the benzyl end-group at 7.29–7.41 ppm (see Figures S2 and S3). Furthermore, UV GPC analysis (λ = 305 nm) confirmed the absence of any unreacted RAFT agent after purification of PLLA_14_-TTC (see Figure S4).

**Figure 1:**
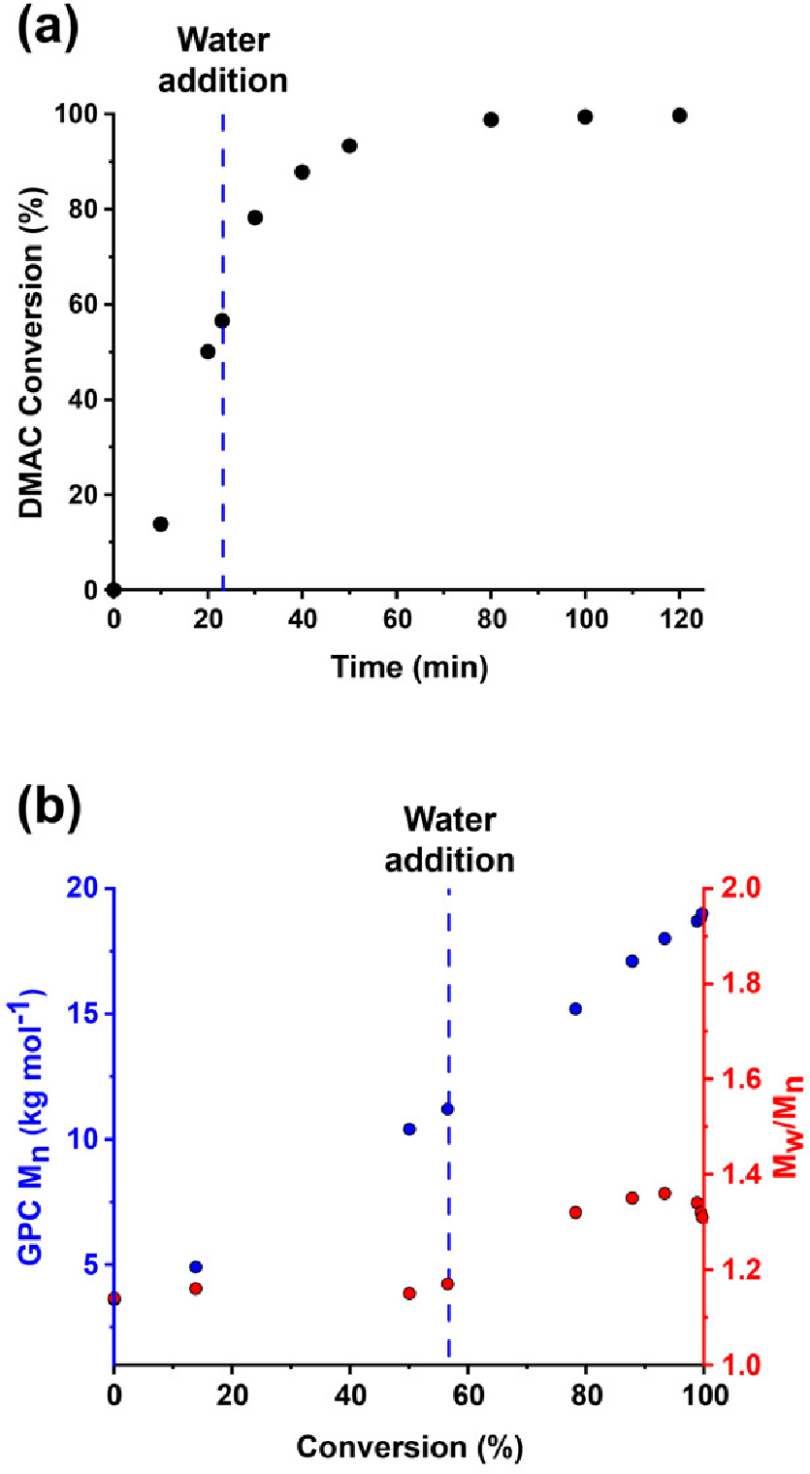
Kinetics of polymerization for the synthesis of PLLA_14_-PDMAC_120_ nanoparticles, where polymerization is initiated in the bulk at 70 °C followed by dilution with degassed deionized water after 23 min (DMAC conversion = 57%) to target 30% w/w solids. Conditions: [PLLA_14_-TTC]/[ACVA] molar ratio = 10. (a) Conversion vs time curve (black data) obtained by ^1^H NMR spectroscopy studies and (b) corresponding molecular weight (*M*_n_, blue points) and dispersity (*M*_w_/*M*_n_, red points) vs conversion data obtained by DMF GPC analysis (expressed relative to a series of PMMA calibration standards). Selected GPC traces are shown in Figure S5.

This PLLA_14_-TTC precursor was then dissolved in *N,N′*-dimethylacrylamide (DMAC) and RAFT polymerization of this vinyl monomer was conducted in the bulk^27,28^ at 70 °C. Once a significant increase in solution viscosity was observed (corresponding to a DMAC conversion of 13–65%), degassed deionized water (preheated to 70 °C) was added to the reaction mixture to target a final solids content of 30% w/w (see Scheme 1). The DMAC polymerization was allowed to proceed for 16 h at 70 °C before quenching by cooling the reaction mixture to 20 °C with concomitant exposure to air. For the synthesis of a PLLA_14_-PDMAC_120_ diblock copolymer, the reaction mixture was periodically sampled, and aliquots were analyzed by GPC and ^1^H NMR spectroscopy to study the polymerization kinetics. After 100 min at 70 °C, 99% DMAC conversion was achieved (Figure 1a).

Remarkably, there was no discernible reduction in the rate of polymerization after dilution of the initial bulk reaction mixture to 30% w/w solids. This is presumably because acrylamides polymerize much faster in aqueous media than in the bulk.^29,30^ GPC analysis indicated a linear increase in molecular weight (*M*_n_) with conversion, suggesting a well-controlled RAFT polymerization. Dispersities always remained less than 1.40 (*M*_w_/*M*_n_ = 1.15 to 1.36) but were nevertheless relatively high for a RAFT polymerization (see Figure 1b). This is attributed to the chemical structure of the RAFT agent, which is not optimized for the well-controlled polymerization of DMAC.

**Figure 2:**
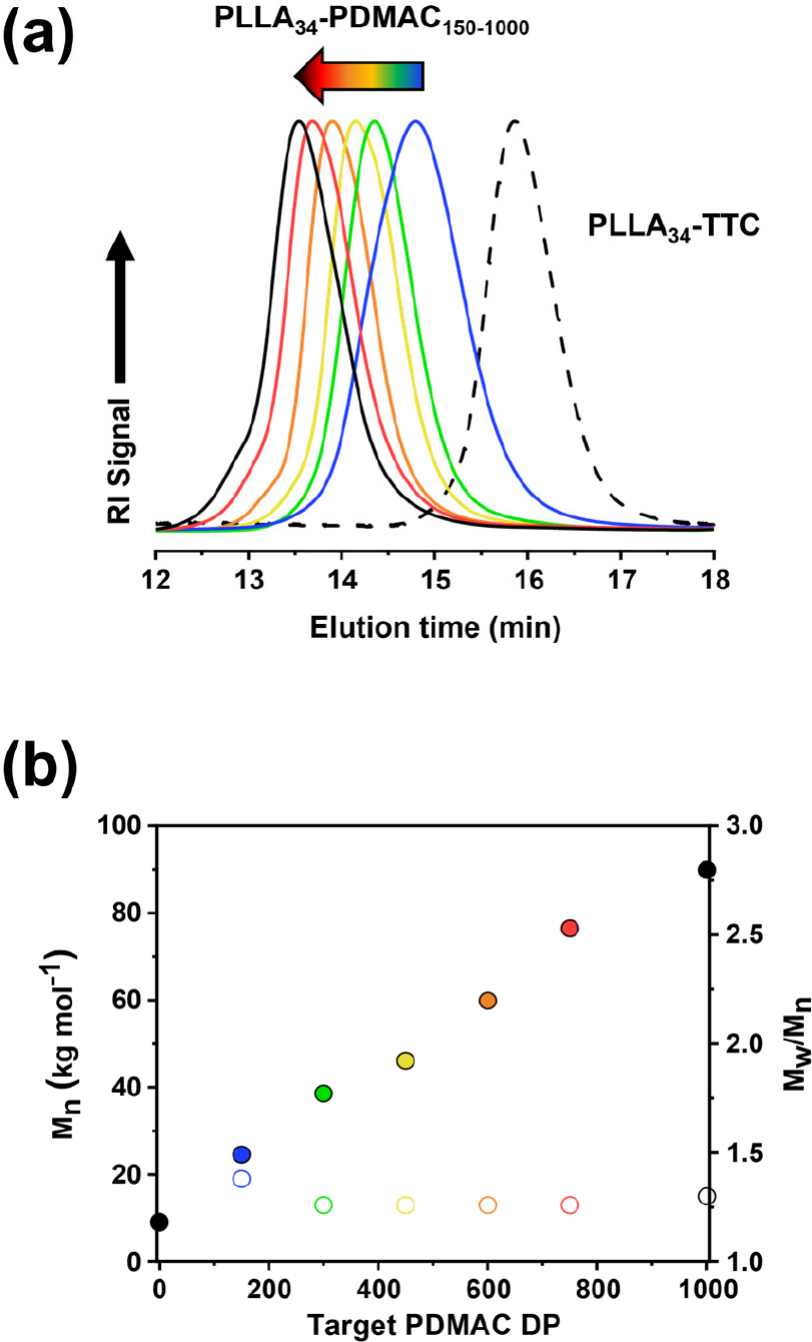
(a) DMF GPC curves (refractive index detector) recorded for a series of PLLA_14_-PDMAC_70–300_ diblock copolymers and the corresponding PLLA_14_-TTC homopolymer. Each copolymer was prepared by reverse sequence aqueous PISA at 70 °C. (b) Corresponding number-average molecular weight (*M*_n_) and dispersity (*M*_w_/*M*_n_) data plotted against the target PDMAC DP.

Indeed, a control experiment conducted using the same hydroxyl-functional RAFT agent to target a PDMAC_100_ homopolymer via RAFT polymerization (initially in the bulk followed by dilution with water to 30% w/w solids at an intermediate conversion of 45%) afforded more than 99% conversion after 16 h at 70 °C. GPC analysis indicated an *M*_n_ of 10.3 kg mol^–1^ and an *M*_w_/*M*_n_ of 1.26, which is somewhat higher than those reported in the literature for such syntheses.^31,32^ The same synthetic protocol was then used to target a series of PLLA_14_-PDMAC_70–300_ diblock copolymers. GPC analysis indicated a linear increase in molecular weight when targeting higher PDMAC DPs with final dispersities as low as 1.28 (Table 1 and Figure 2). UV GPC (λ = 305 nm) studies indicated reasonably high chain extension efficiencies when targeting PDMAC DPs up to 210, suggesting minimal contamination by the PLLA_14_-TTC precursor (<10% residual precursor, see Figure S6).

**Table 1:** Summary of Dilution Times, Intermediate DMAC Conversions, and Molecular Weight Data Obtained for a Series of PLLA_14-34_-PDMAC_70-300_ Nanoparticles and PLLA_34_-PDMAC_150_ Nanoparticles^a^

target diblock copolymer composition	dilution time/min	intermediate DMAC conversion %	*M*_n_/kg mol^–1^	*M*_w_/*M*_n_
PLLA_14_-TTC	N/A	N/A	3.6	1.14
PLLA_14_-PDMAC_70_	32	58	11.5	1.40
PLLA_14_-PDMAC_90_	60	65	13.7	1.44
PLLA_14_-PDMAC_110_	40	50	16.7	1.36
PLLA_14_-PDMAC_130_	30	62	17.5	1.31
PLLA_14_-PDMAC_150_	35	35	19.7	1.35
PLLA_14_-PDMAC_170_	28	40	23.1	1.31
PLLA_14_-PDMAC_190_	32	38	24.7	1.28
PLLA_14_-PDMAC_210_	21	26	28.3	1.29
PLLA_14_-PDMAC_230_	26	43	29.1	1.28
PLLA_14_-PDMAC_300_	29	13	40.4	1.28
PLLA_34_-TTC	N/A	N/A	9.2	1.18
PLLA_34_-PDMAC_150_	20	43	24.4	1.20

a More than 99% DMAC conversion was achieved for all diblock copolymer syntheses.

However, when targeting a PDMAC DP of either 230 or 300, a low molecular weight species corresponding to 15–17% of the total signal was observed. Interestingly, such contamination was not discernible when using a refractive index detector, see Figure 2a. Moreover, ^1^H NMR spectroscopy analysis indicated that more than 99% DMAC conversion was achieved for all syntheses, see Figure S2.

When targeting lower PDMAC DPs of either 50 or 60, problems were encountered when attempting similar syntheses at 70 °C. No precipitation was observed on dilution with water (after 45 or 37 min, respectively). However, DMF GPC analysis using a refractive index detector indicated a bimodal GPC trace in each case, suggesting poor chain extension efficiency (see Figure S7). Furthermore, no high molecular weight species were detected by UV GPC analysis. This indicates that such DMAC polymerizations are poorly controlled because these longer chains do not possess RAFT end-groups (see Figure S7). Moreover, targeting PDMAC DPs below 50 resulted in immediate macroscopic precipitation after dilution with water. Presumably, this is simply because the PDMAC chains that are present when water is added to the reaction mixture are too short to confer effective steric stabilization on the nascent nanoparticles. In contrast, if such syntheses were conducted at 90 °C, then PDMAC DPs as low as 40 could be targeted (see Figure S8).

**Figure 3:**
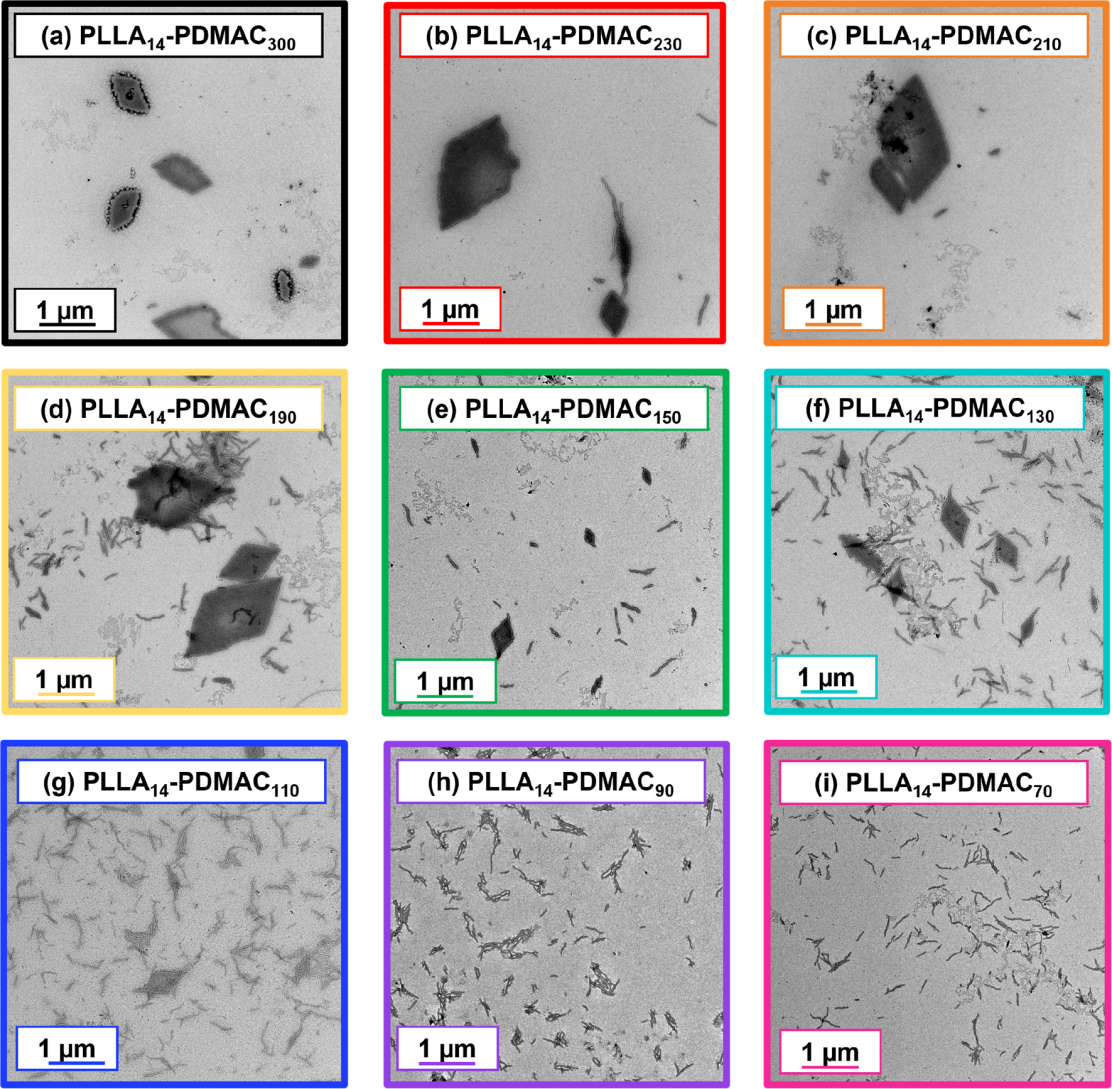
Representative TEM images recorded for dilute aqueous dispersions of a series of PLLA_14_-PDMAC_70–300_ nanoparticles: (a) *x* = 300, (b) 230, (c) 210, (d) 190, (e) 150, (f) 130, (g) 110, (h) 90, and (i) 70.

TEM analysis of the series of PLLA_14_-PDMAC_70–300_ nanoparticles confirmed that the final copolymer morphology depended on the target PDMAC DP. Targeting the highest PDMAC DP of 300 led to the formation of diamond-like platelets (see Figure 3a). In contrast, short rod-like nanoparticles were obtained when targeting the lowest PDMAC DP of 70 (see Figure 3i). Hence our new approach to CDSA enables the efficient formation of highly concentrated aqueous dispersions of anisotropic nanoparticles. We believe this to be an important advance, but the diamond platelets are currently less uniform than those obtained during traditional relatively slow CDSA syntheses performed in dilute solution.^33−35^ However, certain applications such as Pickering emulsifiers and foam stabilizers do not require particularly uniform nanoparticles. In such cases, the ability to prepare anisotropic nanoparticles at high solids concentrations directly in water is likely to be a decisive advantage.

**Figure 4:**
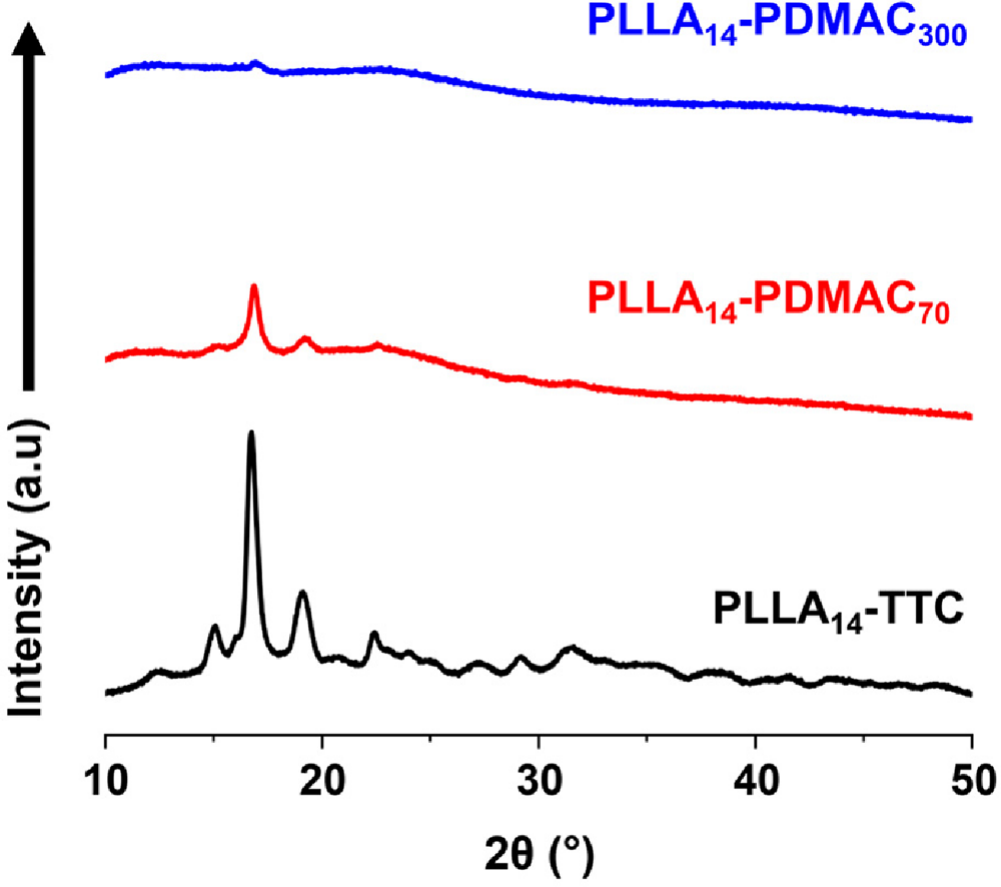
XRD patterns recorded for the PLLA_14_-TTC precursor, freeze-dried PLLA_14_-PDMAC_70_ rod-like nanoparticles, and freeze-dried PLLA_14_-PDMAC_300_ platelets.

Various binary mixtures of these two morphologies were observed for intermediate PDMAC DPs (Figure 3b–g). These observations are in good agreement with literature reports for the self-assembly of PLLA-PDMAC diblock copolymers via CDSA in dilute solution using organic solvents such as methanol or ethanol.^11,33^ It is perhaps worth emphasizing that the design rules for PISA differ significantly from those of CDSA. Our prior reverse sequence PISA syntheses invariably yielded a spherical morphology.^23,36^ This is because such formulations always require a relatively large volume of hydrophilic monomer to solubilize the hydrophobic precursor, which inevitably leads to a relatively long steric stabilizer block. Such diblock copolymer compositions are known to favor the formation of spheres, rather than worms or vesicles.^22^ In contrast, when using crystalline PLLA (or PDLA), it is clear from the CDSA literature that a relatively long steric stabilizer block is essential for the formation of diamond platelets.^33−35^ Hence the judicious combination of reverse sequence PISA with CDSA is an important advance because it provides access to a significantly wider range of copolymer morphologies. The mean % degree of crystallinity, *D*_c_, of the PLLA_14_-PDMAC_300_ platelets and PLLA_14_-PDMAC_70_ rods was determined by X-ray diffraction (XRD), see Figure 4. The diffraction pattern recorded for the PLLA_14_-TTC precursor has a Bragg peak at 17° that corresponds well to that reported in the literature.^37^ The *D*_c_ for this reference sample was 41%. Similarly, *D*_c_ values of 16% and 2% were calculated for PLLA_14_-PDMAC_70_ and PLLA_14_-PDMAC_300_, respectively.

Aqueous electrophoresis studies confirmed that both PLLA_14_-PDMAC_300_ and PLLA_14_-PDMAC_70_ nanoparticles exhibited essentially zero zeta potentials from pH 4 to 9 (see Figure S9), which is consistent with the non-ionic nature of the PDMAC steric stabilizer chains. Differential scanning calorimetry (DSC) studies were performed to examine whether the PLLA_14_-TTC precursor exhibited crystallinity (see Figure S1). As expected, a well-defined glass transition temperature (*T*_g_), crystallization temperature (*T*_c_), and melting temperature (*T*_m_) were observed.^38^ Notably, since the *T*_m_ for PLLA_14_-TTC is around 114 °C, this precursor should be able to direct CDSA given that the *in situ* DMAC polymerization is conducted at 70 °C.^1,2,4−11^

**Figure 5:**
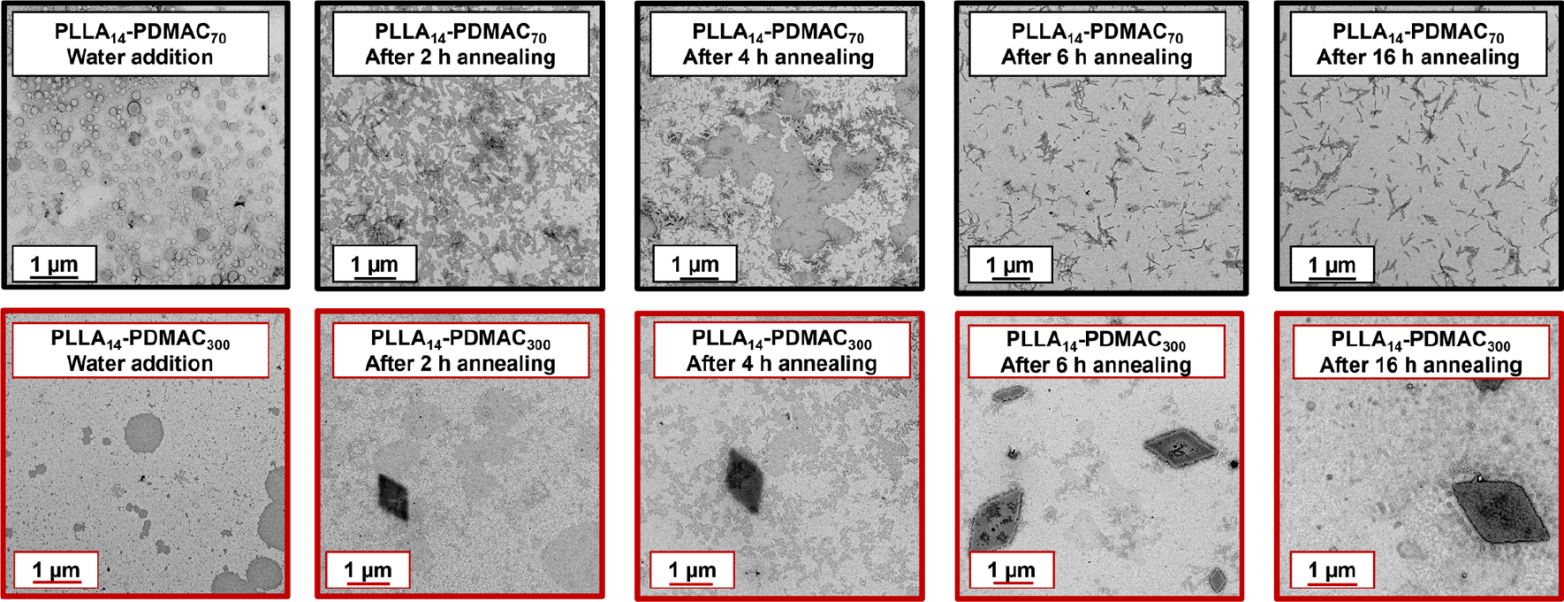
Representative TEM images recorded immediately after’ water addition for the synthesis of PLLA_14_-PDMAC_70_ nanoparticles (images with black outline) and PLLA_14_-PDMAC_300_ nanoparticles (images with red outline) and after annealing for 2, 4, 6, and 16 h.

Given that (i) the DMAC polymerization is initially performed in the bulk and (ii) the DMAC monomer is a good solvent for both PLLA and PDMAC, no *in situ* self-assembly should occur prior to addition of water at a suitable intermediate DMAC conversion. Since water is a bad solvent for PLLA and a good solvent for PDMAC, its addition should result in immediate self-assembly of the growing diblock copolymer chains to form nascent PLLA-core nanoparticles. More specifically, PLLA_14_-PDMAC_70_ and PLLA_14_-PDMAC_300_ reaction mixtures were diluted with water after 32 and 29 min, respectively. The corresponding instantaneous DMAC conversions were 58 and 13%, which correspond to unreacted DMAC/water mass ratios of 1:3 and 1:7, respectively. These concentrated aqueous dispersions were then immediately further diluted to 0.1% w/w for TEM studies, which indicated the formation of nascent spherical aggregates in each case, see Figure 5. However, the final copolymer morphology was either rods or platelets after annealing for 16 h (>99% DMAC conversion). In traditional CDSA syntheses, thermal annealing is important for the growth of the initial copolymer seeds to form the final anisotropic nanoparticles.^39,40^ Accordingly, we examined the effect of annealing PLLA_14_-PDMAC_70–300_ nanoparticles at 70 °C. When targeting PLLA_14_-PDMAC_70_ nanoparticles, ^1^H NMR studies indicated more than 99% DMAC conversion within 2 h at 70 °C. TEM analysis indicated the presence of rod-like nanoparticles at this time point, but some aggregates were also observed, see Figure 5. Annealing at 70 °C for 6 h leads to the disappearance of these aggregates, with no further change in copolymer morphology being observed up to 16 h. The corresponding DLS experiments corroborate the TEM studies: *z*-average diameters of 224 and 194 nm were obtained after 2 and 6 h, respectively. After 16 h, the *z*-average diameter remained almost unchanged at 190 nm.

Similarly, more than 99% DMAC conversion was achieved within 2 h when targeting PLLA_14_-PDMAC_300_ platelets. At this time point, diamond platelets can be observed with a mean long axis of up to 1.3 μm, see Figure 5. Annealing at 70 °C led to the formation of progressively larger diamond platelets: the mean long axis of 1.7 μm observed after 4 h increased to 1.9 μm after 6 h and 2.3 μm after 16 h. Again, DLS studies are consistent with these observations: the apparent *z*-average diameter increased from 74 nm after 2 h to 225 nm after 16 h. It is perhaps worth emphasizing that these DLS values differ significantly from the mean long axes observed by TEM because the Stokes–Einstein equation used to calculate the *z*-average diameter assumes a spherical morphology.^41^ Comparable results were reported by O’Reilly and co-workers when annealing PLLA_48_-PDMAC_1000_ diamond platelets during conventional CDSA syntheses conducted in dilute ethanol at 90 °C.^33^

In another experiment, rod-like nanoparticles were targeted while varying the PLLA DP. Accordingly, PLLA_34_-TTC and PLLA_48_-TTC precursors were prepared via anionic ROP using the same hydroxy-functional RAFT agent and characterized by NMR and GPC analysis (see Figures S10–S12). Subsequently, each precursor was chain-extended in turn with DMAC, initially via bulk polymerization followed by dilution with water at intermediate conversion.

Unfortunately, the PLLA_48_-TTC precursor could not be molecularly dissolved in the DMAC monomer: the initial reaction mixture remained slightly turbid even at 70 °C. Subsequent chain extension when targeting a PDMAC DP of 400 produced an ill-defined copolymer with a dispersity above 1.80. Moreover, increasing the reaction temperature up to 90 °C did not alleviate this problem: the initial reaction mixture was always turbid rather than transparent. Thus the new approach reported herein may be limited to relatively short PLLA DPs, at least when using DMAC monomer. Nevertheless, this is sufficient to provide access to higher order morphologies.

**Figure 6:**
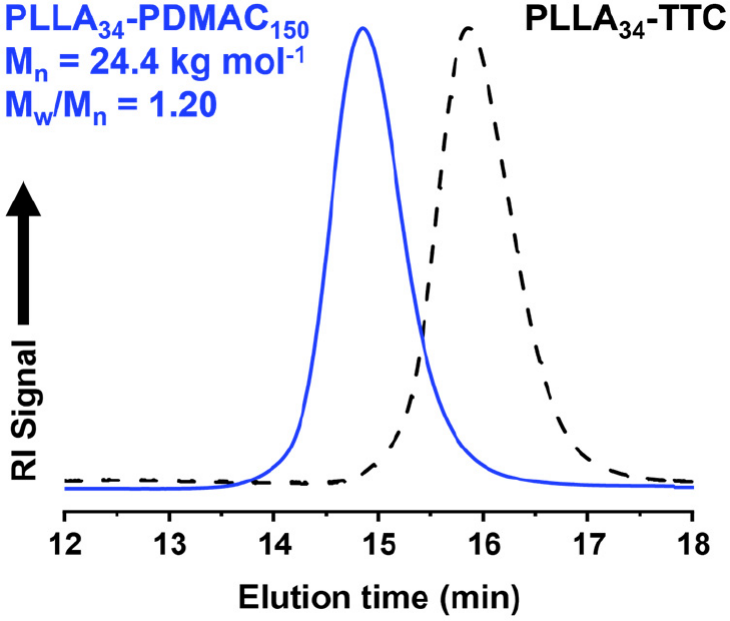
DMF GPC curves (refractive index detector) recorded for a PLLA_34_-PDMAC_150_ diblock copolymer (blue trace) and the corresponding PLLA_34_-TTC homopolymer (black trace). The diblock copolymer was prepared by CDSA using a reverse sequence aqueous PISA formulation at 70 °C.

In contrast, the PLLA_34_-TTC precursor proved to be soluble in DMAC monomer at 70 °C when targeting a DP of 150, and the ensuing polymerization resulted in reasonably well-defined diblock copolymers (*M*_w_/*M*_n_ = 1.20) at a final concentration of 30% w/w solids, see Table 1 and Figure 6. Furthermore, UV GPC analysis confirmed no significant contamination from the PLLA_34_ precursor (see Figure S13). In this case, the final reaction mixture formed a thick paste. XRD analysis indicated *D*_c_ values of 31% and 12% for the PLLA_34_-TTC precursor and PLLA_34_-PDMAC_150_ rod-like nanoparticles, respectively (see Figure S14). TEM analysis of dilute aqueous dispersions of the PLLA_34_-PDMAC_150_ nanoparticles confirmed that a rod-like morphology was formed when targeting a PDMAC DP of 150, see Figure S15.

**Figure 7:**
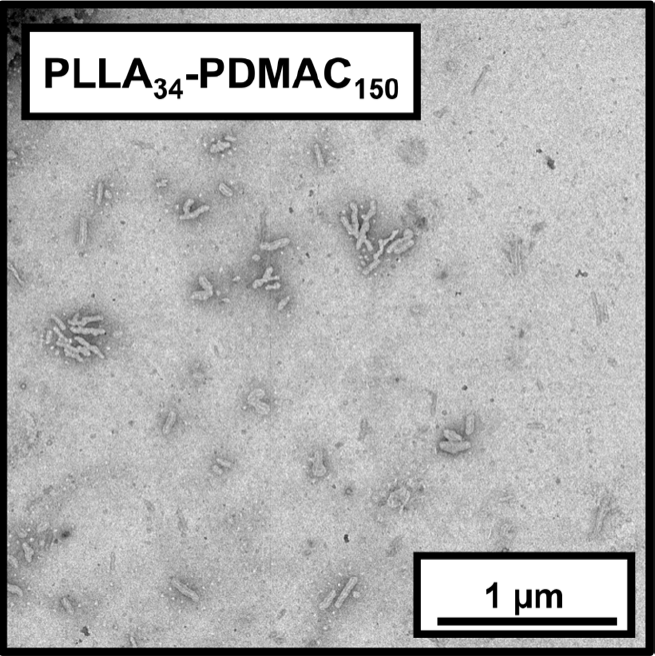
Representative TEM image recorded for a dilute aqueous dispersion of PLLA_34_-PDMAC_150_ nanoparticles prepared at 40% w/w solids.

The effect of varying the final nanoparticle concentration was also examined. Hence PLLA_34_-PDMAC_150_ nanoparticles were prepared at 20 and 40% w/w solids to compare with syntheses targeting 30% w/w solids. In both cases, reasonably well-defined diblock copolymer chains were obtained with dispersities of 1.26 and 1.29 respectively, see Figure S16. Furthermore, the same rod-like morphology was obtained when targeting 40% w/w solids, see Figure 7. In contrast, only ill-defined aggregates were obtained when targeting 20% w/w solids, see Figure S15.

For the same target PLLA_34_-PDMAC_150_ nanoparticles prepared at 30% w/w solids, the time at which water was added to the reaction mixture was systematically varied. No macroscopic precipitation was observed regardless of whether such dilution occurred after 10, 15 or 20 min (which corresponds to intermediate DMAC conversions of 19, 38 and 43%, respectively). Essentially the same diblock copolymer chains were produced when water was added after either 15 or 20 min (see Figure S17) and a rod-like morphology was obtained in each case (see Figures S15b and S18). In contrast, a significantly lower copolymer molecular weight and a higher dispersity were obtained when water was added after 10 min. This suggests the loss of RAFT control under the latter conditions, presumably because the nascent PDMAC chains are too short to confer effective steric stabilization. Water addition was also attempted after 25 min. However, the reaction mixture had solidified at this time point and could not be diluted. Nevertheless, these experiments suggest that our new reverse sequence aqueous PISA plus CDSA protocol can be used to target a range of final copolymer concentrations and is reasonably tolerant of the precise time chosen for water addition.

**Figure 8:**
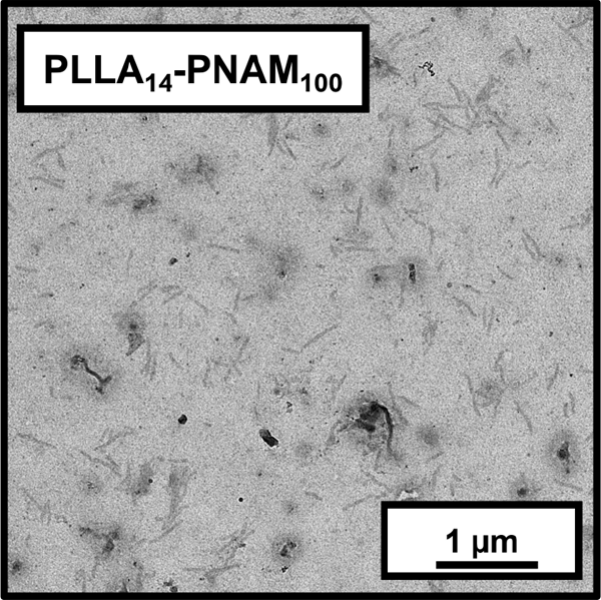
Representative TEM image recorded for a dilute aqueous dispersion of PLLA_14_-PNAM_100_ nanoparticles prepared at 30% w/w solids.

Finally, a second hydrophilic vinyl monomer was briefly examined. Accordingly, *N*-acryloylmorpholine (NAM) was used as an alternative hydrophilic monomer to DMAC when targeting a DP of either 100 or 400 using the PLLA_14_-TTC precursor. Essentially full NAM conversion was achieved (see Figure S19), but copolymer dispersities were broader than those achieved with the DMAC monomer (see Figure S20). More specifically, copolymer dispersities of 1.54 and 1.69 were obtained when targeting PLLA_14_-PNAM_400_ and PLLA_14_-PNAM_100_, respectively. Furthermore, when targeting a PNAM DP of 400, the chain extension efficiency was estimated to be only around 75%. Targeting a mean DP of 100 resulted in rod-like nanoparticles, see Figure 8. However, no well-defined aggregates could be obtained when targeting PLLA_14_-PNAM_400_.

**Figure 9:**
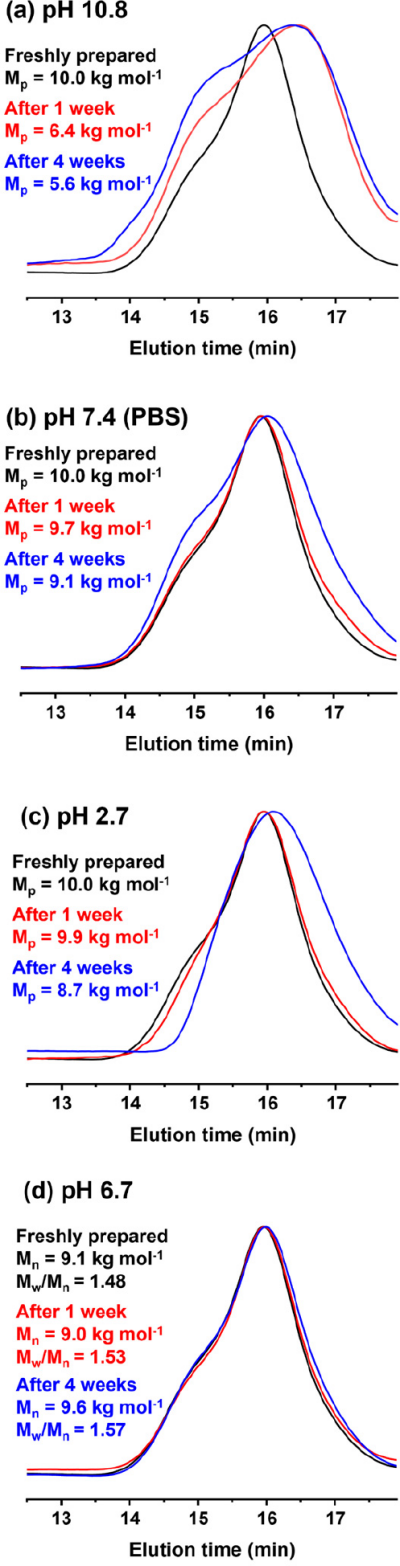
DMF GPC curves (refractive index detector) recorded for freshly prepared PLLA_14_-PDMAC_40_ and after hydrolytic degradation of 1.0% w/w aqueous dispersions of PLLA_14_-PDMAC_40_ nanoparticles after storage at 37 °C for either 1 or 4 weeks at (a) pH 10.8, (b) pH 7.4 (PBS buffer), (c) pH 2.7, and (d) after storage at 20 °C as a 30% w/w aqueous dispersion at pH 6.7 (red curve) for either 1 or 4 weeks.

PLLA is widely recognized to be hydrolytically degradable owing to the cleavable ester bonds within its backbone.^42^ Hence full degradation of this hydrophobic polyester block should yield water-soluble PDMAC chains. Accordingly, degradation studies were undertaken at 37 °C by preparing 1.0% w/w dispersions of PLLA_14_-PDMAC_40_ nanoparticles via dilution using an acidic, basic or neutral aqueous buffer. The rod-like morphology of the PLLA_14_-PDMAC_40_ nanoparticles was confirmed by TEM studies (see Figure S21). GPC was used to assess the extent of the hydrolytic degradation over time. As expected, significant degradation of the diblock copolymer chains was observed within one week for PLLA_14_-PDMAC_40_ nanoparticles stored at pH 10.8 (see Figure 9a). Hydrolytic degradation was also observed in an acidic buffer solution (pH 2.9) and in the presence of a PBS buffer (pH 7.4), albeit at a somewhat slower rate (Figure 9b,c). This is because the base-catalyzed hydrolysis of ester bonds is known to be faster than acid-catalyzed hydrolysis.^43^ However, aging a 30% w/w aqueous dispersion of the same nanoparticles in deionized water (pH 6.7) at 20 °C led to minimal discernible degradation over four weeks (see Figure 9d). Furthermore, these PLLA_14_-PDMAC_40_ nanoparticles retained their colloidal stability over the same time period (see Figure S22).

DLS studies of these rod-like PLLA_14_-PDMAC_40_ nanoparticles in mildly basic solution (pH 10.8) confirmed a significant reduction in light scattering count rate from 56,000 to 5000 kcps within four weeks. This suggests that the original nanoparticles are converted into water-soluble PDMAC_40_ chains. Similarly, GPC analysis confirmed that the diblock copolymer *M*_n_ was reduced from 9.6 to 5.7 kg mol^–1^ within 24 h during an accelerated aging experiment performed at 60 °C in the presence of 5.0% w/w aqueous KOH, indicating complete degradation of the PLLA block. Concomitant DLS studies indicated a substantial reduction in the light scattering count rate from 60,000 to 500 kcps, while the number-average particle diameter was reduced from 160 to 2.8 nm. This is consistent with nanoparticle dissolution to form water-soluble PDMAC chains. One reviewer of this manuscript has pointed out that this non-degradable component comprises the majority of the mass of the original nanoparticles.

## Conclusions

In summary, reverse sequence PISA has been combined with CDSA to enable the efficient preparation of 30% w/w aqueous dispersions of highly anisotropic hydrolytically degradable PLLA_14_-PDMAC_*x*_ diblock copolymer nanoparticles. The crystalline nature of the hydrophobic PLLA block produces either diamond-like platelets (e.g., PLLA_14_-PDMAC_300_) or short rod-like particles (e.g., PLLA_14_-PDMAC_70_). ^1^H NMR spectroscopy analysis confirms that approximately 99% DMAC conversion is achieved within 100 min at 70 °C when targeting PLLA_14_-PDMAC_120._ A linear increase in molecular weight with increasing conversion is observed, but relatively broad molecular weight distributions are observed owing to the use of a suboptimal RAFT agent. Nevertheless, *M*_w_/*M*_n_ values do not exceed 1.44 for syntheses conducted at 70 °C, and this minor technical problem should be readily addressable in the future. Given that these anisotropic nanoparticles are prepared directly in concentrated aqueous media, this is the first truly viable route for their industrial manufacture. Furthermore, preliminary data suggest that a PLLA_34_ precursor and an alternative hydrophilic vinyl monomer (NAM) can be employed for such syntheses. Importantly, such nanoparticles are susceptible to hydrolytic degradation. We anticipate that this highly convenient new synthetic protocol should aid the evaluation of these anisotropic nanoparticles as next-generation sustainable Pickering emulsifiers^34^ and foam stabilizers.^44^

## Supporting Information Available

Full experimental details, including materials, instrumentation, analytical protocols, and synthetic protocols, summary table of reagent quantities used for polymerizations, additional NMR, DSC, and GPC data for the PLLA precursor, further GPC, NMR, TEM, DLS, and aqueous electrophoresis data for the PLLA-based diblock copolymers, and GPC and DLS hydrolytic degradation data for control experiments (PDF)
