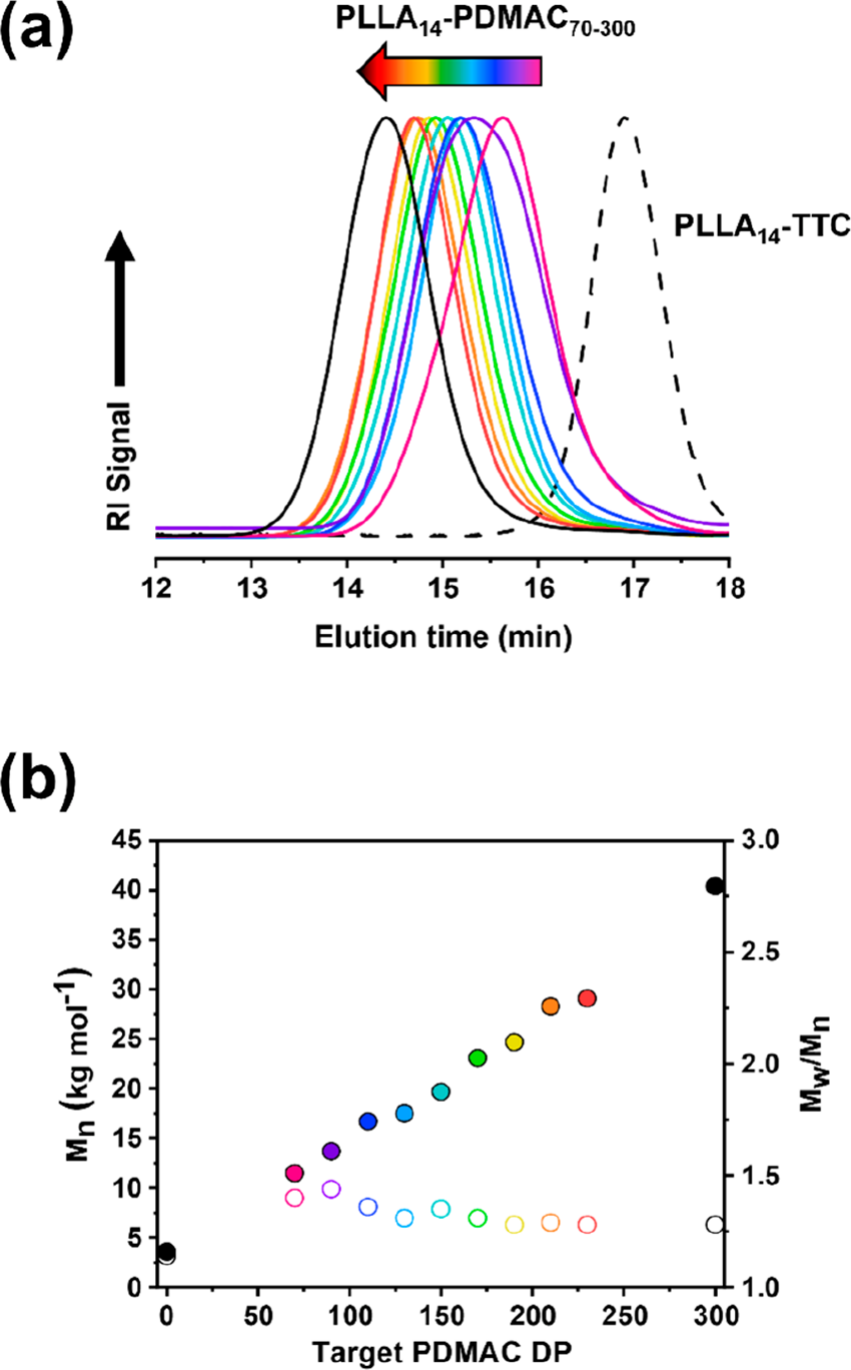# Correction to “Combining Crystallization-Driven Self-Assembly with Reverse Sequence Polymerization-Induced Self-Assembly Enables the Efficient Synthesis of Hydrolytically Degradable Anisotropic Block Copolymer Nano-objects Directly in Concentrated Aqueous Media”

**DOI:** 10.1021/jacs.5c11548

**Published:** 2025-08-13

**Authors:** Matthew A. H. Farmer, Osama M. Musa, Steven P. Armes

The authors regret that Figure 2 in our original manuscript was inadvertently replaced with an incorrect version at the galley proof stage. The correct version of Figure 2 is provided below. The corrected Figure 2 and data therein do not alter the conclusions of this publication.