# Supplementary material for: Combining Crystallization-Driven Self-Assembly with Reverse Sequence Polymerization-Induced Self-Assembly Enables the Efficient Synthesis of Hydrolytically Degradable Anisotropic Block Copolymer Nano-objects Directly in Concentrated Aqueous Media
Source: J Am Chem Soc. 2024 Jun 6;146(24):16926–34. doi: 10.1021/jacs.4c06299 (PMC11191691; doi:10.1021/jacs.4c06299)
Supplement: Supplementary file 1 — ja4c06299_si_001.pdf [file ja4c06299_si_001.pdf]

Supporting Information for:

Combining crystallization-driven self-assembly with reverse sequence polymerization-induced self-assembly enables efficient synthesis of hydrolytically degradable anisotropic block copolymer nano-objects directly in concentrated aqueous media

Matthew A. H. Farmer,[a] Osama M. Musa,[b] and Steven P. Armes[a, *]

a. Dainton Building, Department of Chemistry, University of Sheffield, Sheffield, S3 7HF, UK.

b. Ashland Specialty Ingredients, 1005 US 202/206, Bridgewater, New Jersey, 08807, USA.

*Corresponding author: s.p.armes@sheffield.ac.uk

Contents

Experimental...S2
Materials...S2
Characterization Techniques...S2
Synthetic Protocols...S3
Supplementary Characterization Data...S7
References...S17

Experimental

Materials

All reagents were used as received, unless stated otherwise. 4,4′-Azobis(4-cyanopentanoic acid) (ACVA; 98%), *N,N'*-dimethylacrylamide (DMAC; 99%), anhydrous magnesium sulfate and lithium bromide were purchased from Sigma-Aldrich (Dorset, UK). Ammonia solution (28%), *N*-acryloyl morpholine (NAM; >97%) and 4-(dimethylamino)pyridine (DMAP) were purchased from Alfa Aesar (Heysham, UK). *N,N'*-Dimethylformamide (DMF) was purchased from VWR (Leicestershire, UK). Methanol, ammonium chloride, potassium hydroxide, Oxoid™ phosphate-buffered saline tablets hydrochloric acid (38%) and *L*-lactide (>98%) were purchased from Fisher Scientific (Loughborough, UK). The latter monomer was recrystallized from toluene before use. Deuterated dichloromethane (99.8%) was purchased from Goss Scientific Instruments Ltd (Cheshire, UK). Anhydrous dichloromethane was obtained from an in-house Grubbs purification solvent system. Benzyl 2-hydroxyethyl trithiocarbonate (BHETTC) was prepared using a literature protocol.[1] Deionized water was dispensed from an Elgastat Option 3A water purification system with a resistivity of 15 MΩ cm.

Characterization Techniques

^{1}H Nuclear Magnetic Resonance Spectroscopy. All spectra were recorded in CD_2Cl_2 using a 400 MHz Bruker Avance-400 spectrometer operating at 298 K. Sixteen scans were averaged per spectrum. Aqueous copolymer dispersions were dried with anhydrous magnesium sulfate before passing through a 0.20 µm filter. DMAC conversions were calculated by comparing the integrated vinyl proton signals at 6.63, 6.23 and 5.66 ppm to the aromatic signals assigned to the benzyl end-group at 7.07-7.39 ppm.

Gel Permeation Chromatography. All chromatograms were recorded at 60°C with an Agilent 1260 Infinity GPC system comprising two Agilent PL-gel 5 µm Mixed-C columns and a guard column connected in series, a differential refractive index detector and a UV detector (λ = 305 nm) and a flow rate of 1.0 ml min^{-1}. Agilent GPC/SEC software was used to determine the number-average molecular weight (M_n), weight-average molecular weight (M_w) and dispersity (M_w/M_n) for each (co)polymer. Unless otherwise stated, HPLC-grade DMF containing 10 mM LiBr was used as the eluent. The system was calibrated with a series of twelve near-monodisperse poly(methyl methacrylate) standards with M_p values ranging from 800 g mol^{-1} to 2,200,000 g mol^{-1}. Samples were prepared for GPC analysis by diluting to 1.0% w/w using the GPC eluent.

Dynamic Light Scattering. All experiments were conducted at 20 °C using a Malvern Instruments Zetasizer Nano ZS instrument equipped with a 4 mW He−Ne laser (λ = 633 nm), where scattered light was detected at 173 ° with an avalanche photodiode detector. Samples were prepared for DLS analysis by diluting to 0.1% w/w with deionized water prior to analysis. Samples of PLLA$_{34}$-PDMAC$_{150}$ particles diluted to 0.1% w/w were stirred for 24 h to ensure complete dispersion. All samples were equilibrated in the DLS spectrometer for five minutes prior to analysis to allow for thermal equilibration. The mean z-average particle diameter (D_z) and polydispersity index (PDI) were averaged over two consecutive runs consisting of ten measurements each.

Transmission Electron Microscopy. An FEI Tecnai Spirit 2 microscope equipped with an Orius SC1000B camera operating at 80 kV was used for imaging. A plasma glow discharge was used to treat copper/palladium grids (Agar Scientific, UK) that had been coated in-house with a thin film of amorphous carbon for 30 seconds to generate a hydrophilic surface. Subsequently, a 10 μL droplet of freshly diluted 0.1% w/w aqueous copolymer dispersion was placed on a hydrophilic grid for 1 min, then blotted to remove excess sample. A 10 μL droplet of aqueous uranyl formate solution (0.75% w/v) was used to negatively stain the samples for a further 25 seconds before the grid was carefully blotted to remove excess stain. Each grid was carefully dried with the aid of a vacuum hose.

Aqueous Electrophoresis. To prepare copolymer dispersions for analysis, 1 mM KCl was used to dilute aqueous copolymer dispersions to 0.1% w/w. A Malvern Instruments Zetasizer Nano ZS instrument was used for electrophoretic characterization and mobilities were determined at 20 °C. Solution pH adjustments were performed using either 0.1 M NaOH or 0.1 M HCl as required. Zeta potentials were calculated from the Henry equation using the Smoluchowski approximation.

Differential Scanning Calorimetry. Measurements were performed using a TA DSC25 Discovery series instrument operating from 0 to 150 °C at a heating/cooling rate of 10 °C min^{-1} using aluminum T_{zero} pans and standard lids. Instrument calibration was performed using an indium standard. All DSC analyses involved three heating/cooling cycles.

X-Ray Diffraction. Prior to XRD analysis, all aqueous dispersions were freeze-dried overnight. Powder X-RD analysis was performed using Cu-Kα radiation (40 kV, 40 mA) on a Bruker D8 ADVANCE X-ray powder diffractometer equipped with a motorized divergence slit for Bragg-Brentano geometry and a high-resolution energy-dispersive Lynxeye XE detector. The crystallinity of the PLLA component of each diblock copolymer was calculated using Igor 8.04 multipeak fit software. The crystallinity for the $PLLA_{14}$-TTC precursor was calculated using diffrac.eva v6.1 software.

Synthetic Protocols

Synthesis of trithiocarbonate-capped poly(L-lactide)

Synthesis of the $PLLA_{14}$-TTC precursor was performed according to a literature protocol.[2] All glassware was dried in a 200°C oven overnight prior to use. To a flame-dried Schlenk flask charged with a magnetic stirrer bar, *L*-lactide (5.91 g, 41.0 mmol; target DP = 20), DMAP (0.75 g, 6.12 mmol), BHETTC RAFT agent (0.50 g, 0.45 mL, 2.05 mmol) and dry CH_2Cl_2 (30 mL) were added under a flow of nitrogen gas. The reaction mixture was stirred at 35°C under a nitrogen atmosphere for 18 h. The resulting solution was precipitated into methanol that had been pre-chilled in a -20°C freezer overnight and filtered. The solid product was then washed with excess methanol and dried under vacuum. ^{1}H NMR spectroscopy was used to estimate the mean degree of polymerization (DP) of the PLLA-TTC precursor by end-group analysis (the integrated PLLA backbone signal at 5.10-5.26 ppm was compared to the aromatic signal assigned to the BHETTC initiator at

7.07-7.39 ppm. The final *L*-lactide conversion was 70%, which corresponded to a mean DP of 14. For the analogous syntheses of $PLLA_{34}$-TTC and $PLLA_{48}$-TTC, essentially the same protocol was followed, except that the mass of BHETTC RAFT agent was reduced to 0.20 g and 0.14 g, respectively and the mass of the DMAP was reduced to 0.30 g and 0.21 g respectively.

RAFT polymerization of DMAC in the bulk using $PLLA_{14}$-TTC with subsequent dilution to 30% w/w solids with water at a suitable intermediate DMAC conversion

A 14 mL vial was charged with $PLLA_{14}$-TTC (0.10 g, 0.044 mmol), DMAC (1.31 g, 13.3 mmol, target DP = 300), ACVA (1.2 mg, 4.4 µmol, [TTC]/[ACVA] molar ratio = 10.0) and a magnetic stirrer bar and sealed with a rubber septum. The contents of this vial were deoxygenated with a stream of dry nitrogen gas for 30 min while the vial was immersed in an ice bath to prevent evaporation. The vial was then allowed to warm to room temperature for 10 min before immersion in an oil bath set at 70 °C. The reaction mixture was stirred magnetically and monitored by visual inspection. When the reaction mixture became much more viscous after 32 min, deoxygenated deionized water (3.30 mL, preheated to 70 °C, targeting 30% w/w solids) was added using a degassed syringe/needle. At this point, the reaction vial was removed from the oil bath and subjected to vortex mixing for 2 min to ensure a homogeneous solution, then returned to the oil bath. At this time point, the reaction mixture was sampled for ^{1}H NMR spectroscopy analysis, which indicated an instantaneous DMAC conversion of 13% (PDMAC DP ~ 39). The DMAC polymerization was allowed to proceed for 16 h prior to quenching by exposing the reaction mixture to air while cooling to 20 °C. A final DMAC conversion of more than 99% was indicated by ^{1}H NMR studies. For analogous syntheses in which the target PDMAC and PLLA DP was varied, the reagent quantities and volume of added water were adjusted accordingly (see **Table S1**).

RAFT polymerization of NAM in the bulk using $PLLA_{14}$-TTC with subsequent dilution to 30% w/w solids with water at a suitable intermediate NAM conversion

A 14 mL vial was charged with $PLLA_{14}$-TTC (0.10 g, 0.044 mmol), NAM (2.50 g, 17.7 mmol, target DP = 400), ACVA (1.2 mg, 4.4 µmol, [TTC]/[ACVA] molar ratio = 10.0) and a magnetic stirrer bar and sealed with a rubber septum. The contents of this vial were deoxygenated with a stream of dry nitrogen gas for 30 min while the vial was immersed in an ice bath to prevent evaporation. The vial was then allowed to warm to room temperature for 10 min before immersion in an oil bath set at 70 °C. The reaction mixture was stirred magnetically and monitored by visual inspection. When the reaction mixture became much more viscous after 10 min, deoxygenated deionized water (6.06 mL, preheated to 70 °C, targeting 30% w/w solids) was added using a degassed syringe/needle. At this point, the reaction vial was removed from the oil bath and subjected to vortex mixing for 2 min to ensure a homogeneous solution, then returned to the oil bath. At this time point, the reaction mixture was sampled for ^{1}H NMR spectroscopy analysis, which indicated an instantaneous NAM conversion of 22% (PNAM DP ~ 88). The NAM polymerization was allowed to proceed for 16 h prior to quenching by exposing the reaction mixture to air while cooling to 20 °C. A final NAM conversion of more than 99% was indicated by ^{1}H NMR studies. For analogous syntheses in which a PNAM DP of 100 was targeted, the reagent quantities and volume of added water were adjusted accordingly. Water was added a 15 min at an intermediate NAM conversion of 38% (PNAM DP ~ 38).

Hydrolytic degradation of diblock copolymer nanoparticles in aqueous solution

Acidic, basic and phosphate-buffered saline (PBS) buffer solutions were prepared as follows. Potassium dihydrogen phosphate (10.00 g, 0.073 mol) was dissolved in deionized water (80 mL). Then the solution pH was adjusted with 0.1 M HCl and made up to 100 mL with deionized water to provide a final solution pH of 2.7. Ammonium chloride (6.80 g, 0.127 mol) was dissolved in 28% aqueous ammonia solution (100 mL) to afford a final solution pH of 10.8. A single Oxoid PBS tablet was dissolved in deionized water (100 mL) and 0.1 M HCl was used to adjust the solution pH to pH 7.4. A 30% w/w aqueous dispersion of $PLLA_{14}$-$PDMAC_{40}$ nanoparticles was diluted to 1.0% w/w using each of the above aqueous solutions in turn. The resulting three aqueous copolymer dispersions were stirred at 37 ^{o}C for two weeks and sampled periodically for DMF GPC analyses (refractive index detector). For accelerated degradation experiments, potassium hydroxide (0.50 g, 8.91 mmol) was dissolved in deionized water (10 mL). The solution pH was measured to be 14. This alkaline solution was then used to dilute a 30% w/w aqueous dispersion of $PLLA_{14}$-$PDMAC_{40}$ nanoparticles to 1.0% w/w and this dilute dispersion was then stirred at 60 ^{o}C.

Table S1. Summary of reagent quantities used for the initial bulk polymerization of DMAC using a $PLLA_{14}$-TTC or $PLLA_{34}$-TTC precursor, with subsequent dilution with addition of deoxygenated deionized water at 70ºC. Instantaneous conversions at the time of dilution were determined via ^{1}H NMR spectroscopy. [a]This synthesis was conducted at 90ºC.

Entry Number	Target copolymer composition	Mass of $PLLA_{14}$-TTC grams	Mass of DMAC grams	Mass of ACVA initiator mg	Volume of added water mL	Dilution time min	Intermediate DMAC conversion %
1	$PLLA_{14}$-$PDMAC_{300}$	0.10 (0.042 mmol)	1.31 (13.3 mmol)	1.2 (4.4 µmol)	3.30	32	13
2	$PLLA_{14}$-$PDMAC_{230}$	0.10 (0.042 mmol)	1.01 (10.2 mmol)	1.2 (4.4 µmol)	2.59	60	43
3	$PLLA_{14}$-$PDMAC_{210}$	0.10 (0.042 mmol)	0.92 (9.29 mmol)	1.2 (4.4 µmol)	2.38	40	26
4	$PLLA_{14}$-$PDMAC_{190}$	0.10 (0.042 mmol)	0.83 (8.40 mmol)	1.2 (4.4 µmol)	2.18	30	38
5	$PLLA_{14}$-$PDMAC_{170}$	0.10 (0.042 mmol)	0.75 (7.52 mmol)	1.2 (4.4 µmol)	1.97	35	40
6	$PLLA_{14}$-$PDMAC_{150}$	0.10 (0.042 mmol)	0.66 (6.63 mmol)	1.2 (4.4 µmol)	1.77	28	35
7	$PLLA_{14}$-$PDMAC_{130}$	0.10 (0.042 mmol)	0.57 (5.75 mmol)	1.2 (4.4 µmol)	1.57	32	62
8	$PLLA_{14}$-$PDMAC_{120}$	0.10 (0.042 mmol)	0.53 (5.31 mmol)	1.2 (4.4 µmol)	1.46	23	57
9	$PLLA_{14}$-$PDMAC_{110}$	0.10 (0.042 mmol)	0.48 (4.86 mmol)	1.2 (4.4 µmol)	1.36	21	50
10	$PLLA_{14}$-$PDMAC_{90}$	0.10 (0.042 mmol)	0.39 (3.98 mmol)	1.2 (4.4 µmol)	1.16	26	65
11	$PLLA_{14}$-$PDMAC_{70}$	0.10 (0.042 mmol)	0.31 (3.10 mmol)	1.2 (4.4 µmol)	0.95	29	58
12[a]	$PLLA_{14}$-$PDMAC_{60}$	0.10 (0.042 mmol)	0.26 (2.65 mmol)	1.2 (4.4 µmol)	0.85	4	51
13[a]	$PLLA_{14}$-$PDMAC_{50}$	0.10 (0.042 mmol)	0.22 (2.21 mmol)	1.2 (4.4 µmol)	0.75	6	39
14[a]	$PLLA_{14}$-$PDMAC_{40}$	0.10 (0.042 mmol)	0.18 (1.77 mmol)	1.2 (4.4 µmol)	0.65	7	51
15	$PLLA_{34}$-$PDMAC_{150}$	0.10 (0.019 mmol)	0.29 (2.92 mmol)	0.5 (2.0 µmol)	1.57	16	41
16	$PLLA_{34}$-$PDMAC_{150}$	0.10 (0.019 mmol)	0.29 (2.92 mmol)	0.5 (2.0 µmol)	0.91	20	43
17	$PLLA_{34}$-$PDMAC_{150}$	0.10 (0.019 mmol)	0.29 (2.92 mmol)	0.5 (2.0 µmol)	0.59	13	36

Supplementary Characterization Data

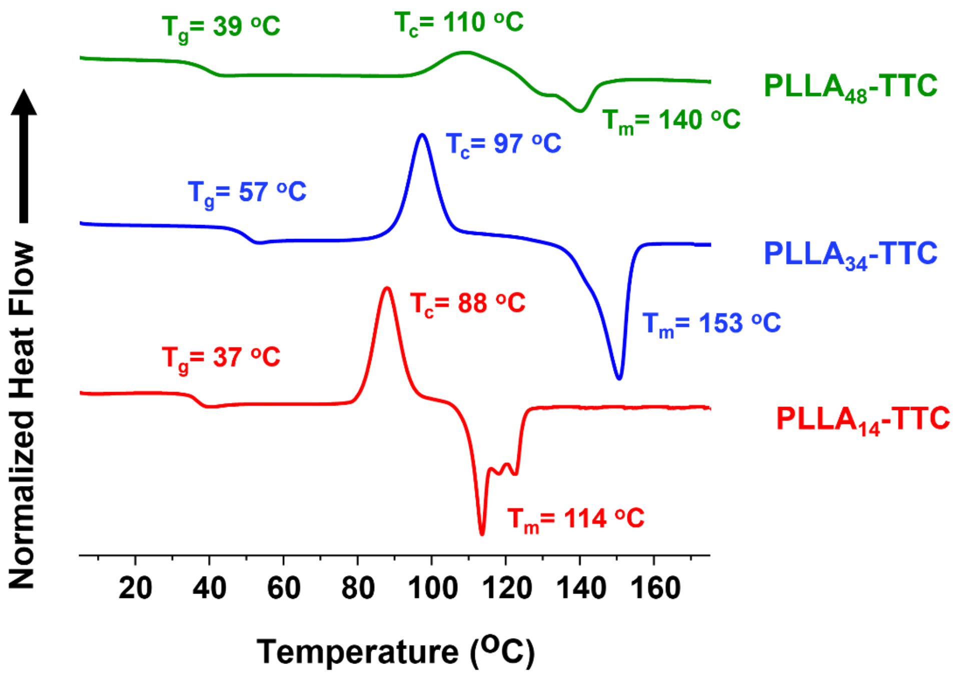

Figure S1. DSC curve recorded for a PLLA$_{14}$-TTC (red trace), PLLA$_{34}$-TTC (blue trace) and PLLA$_{48}$-TTC (green trace) precursor at a heating rate of 10°C per min.

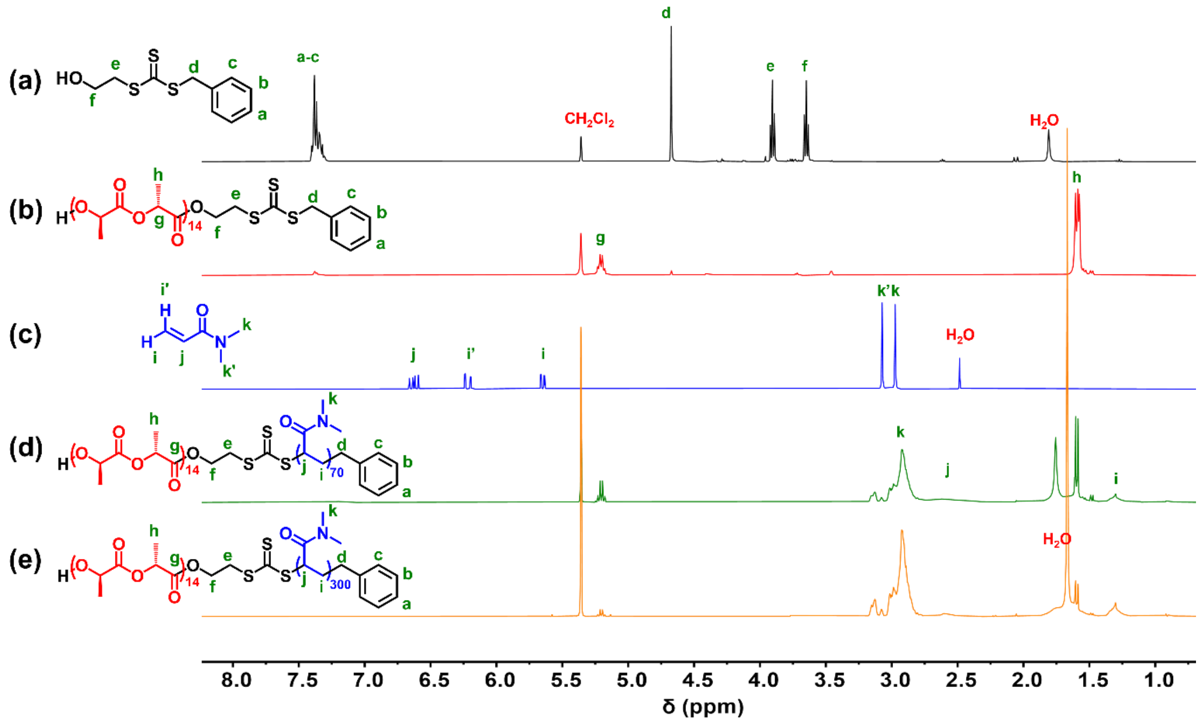

Figure S2. ^{1}H NMR spectra (CD_2Cl_2) recorded for (a) the hydroxy-functional RAFT agent, (b) the PLLA$_{14}$-TTC precursor (c) DMAC monomer, (d) a PLLA$_{14}$-PDMAC$_{70}$ and (e) a PLLA$_{14}$-PDMAC$_{300}$ diblock copolymer.

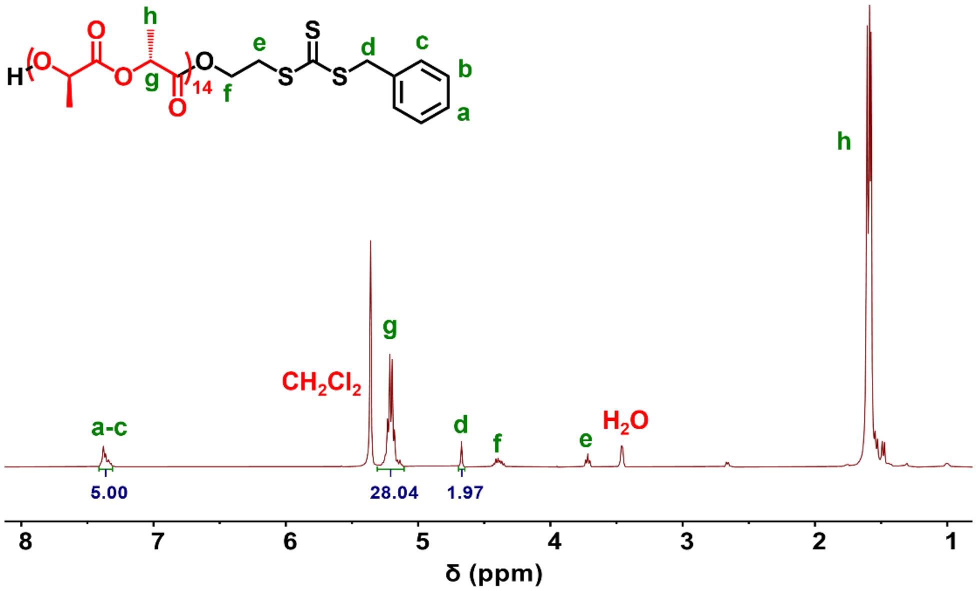

Figure S3. Assigned ^{1}H NMR spectrum (CD_2Cl_2) recorded for a $PLLA_{14}$-TTC precursor, which was prepared via DMAP-catalyzed anionic ring-opening polymerization of *L*-lactide using a hydroxy-functional RAFT agent as an initiator.

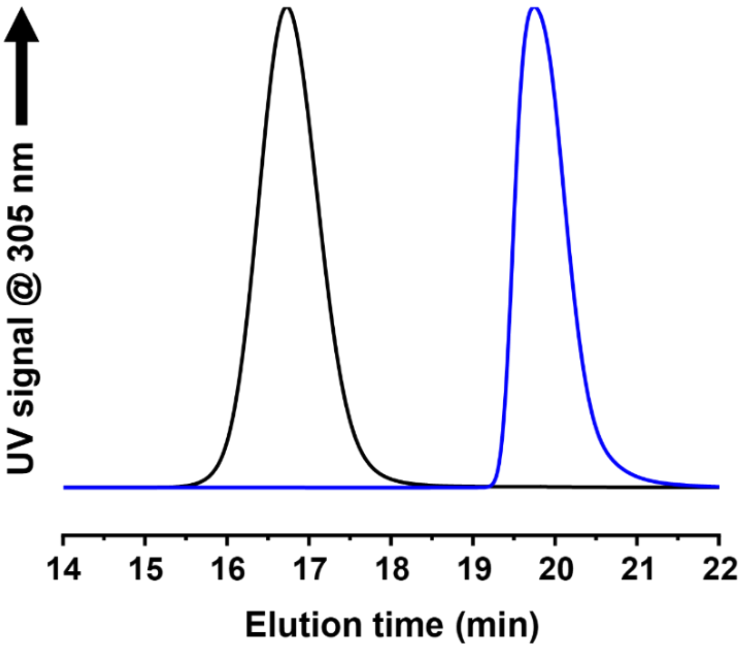

Figure S4. DMF UV GPC curves (λ = 305 nm) recorded for $PLLA_{14}$-TTC (black curve) and the hydroxy-functional RAFT agent (blue curve). For GPC analysis of the RAFT agent, the DMF eluent contained 1% glacial acetic acid but no LiBr. For GPC analysis of the $PLLA_{14}$-TTC, the DMF eluent contained 10 mM LiBr but no glacial acetic acid.

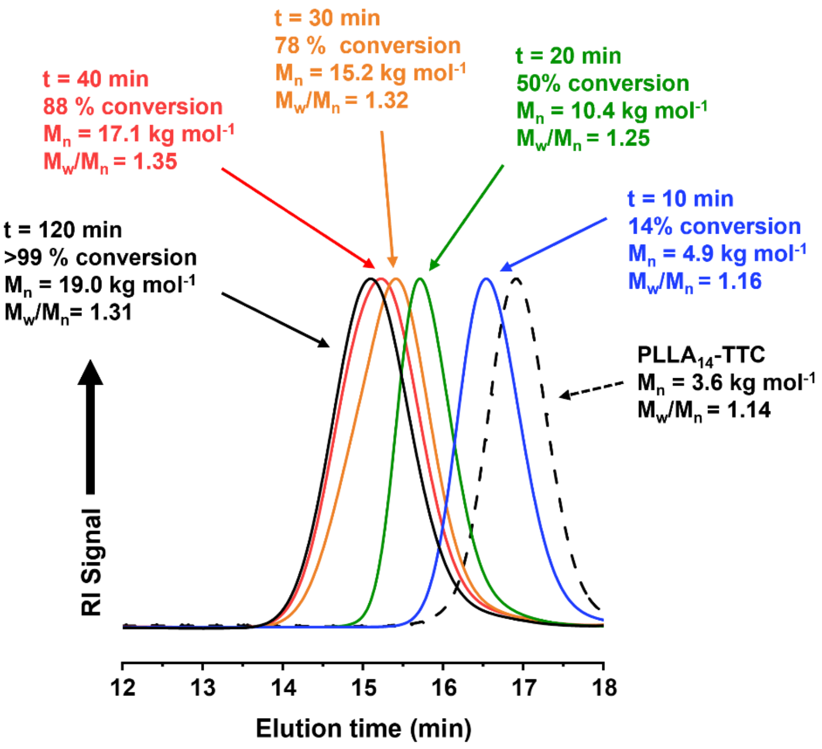

Figure S5. Selected DMF GPC curves (refractive index detector) recorded during the synthesis of $PLLA_{14}$-$PDMAC_{120}$ nanoparticles prepared at 70 °C. Initially, the RAFT polymerization of DMAC was conducted in the bulk, with subsequent dilution to 30% w/w solids using deoxygenated deionized water after 23 min (which corresponds to approximately 57% DMAC conversion). Conditions: [$PLLA_{14}$-TTC]/[ACVA] molar ratio = 10.0.

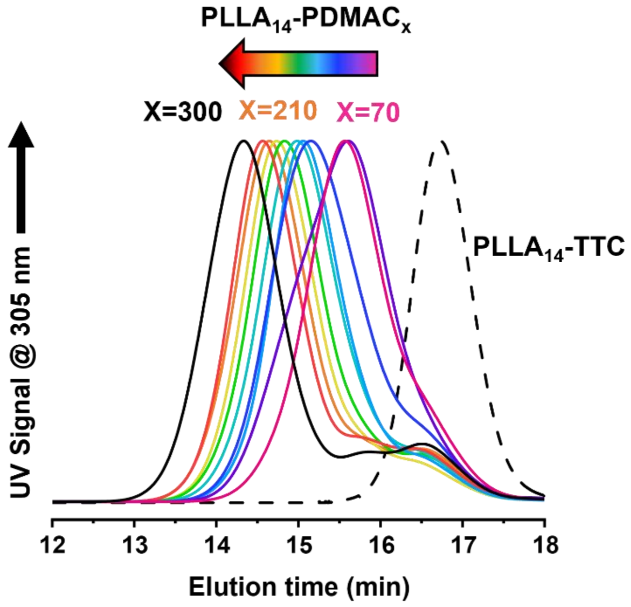

Figure S6. DMF UV GPC curves (λ = 305 nm) recorded for a series of $PLLA_{14}$-$PDMAC_{70-300}$ diblock copolymers and a $PLLA_{14}$-TTC homopolymer precursor. Copolymers were prepared by *reverse sequence* aqueous PISA at 70°C.

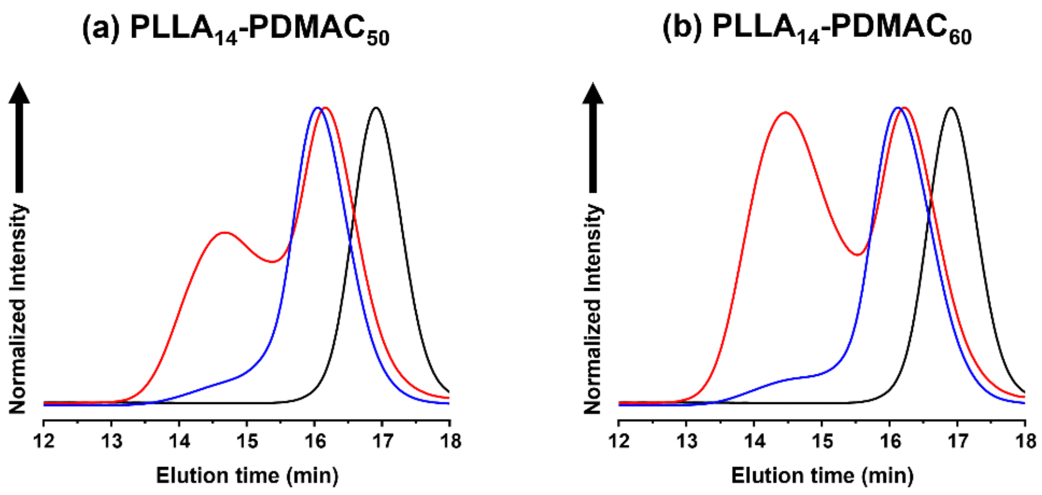

Figure S7. DMF GPC curves (refractive index detector = red trace, UV detector @ 305 nm = blue trace) recorded for (a) $PLLA_{14}$-$PDMAC_{50}$ and (b) $PLLA_{14}$-$PDMAC_{60}$ diblock copolymers and a $PLLA_{14}$-TTC homopolymer (black trace, RI detection). Each copolymer was prepared by *reverse sequence* aqueous PISA at 70°C.

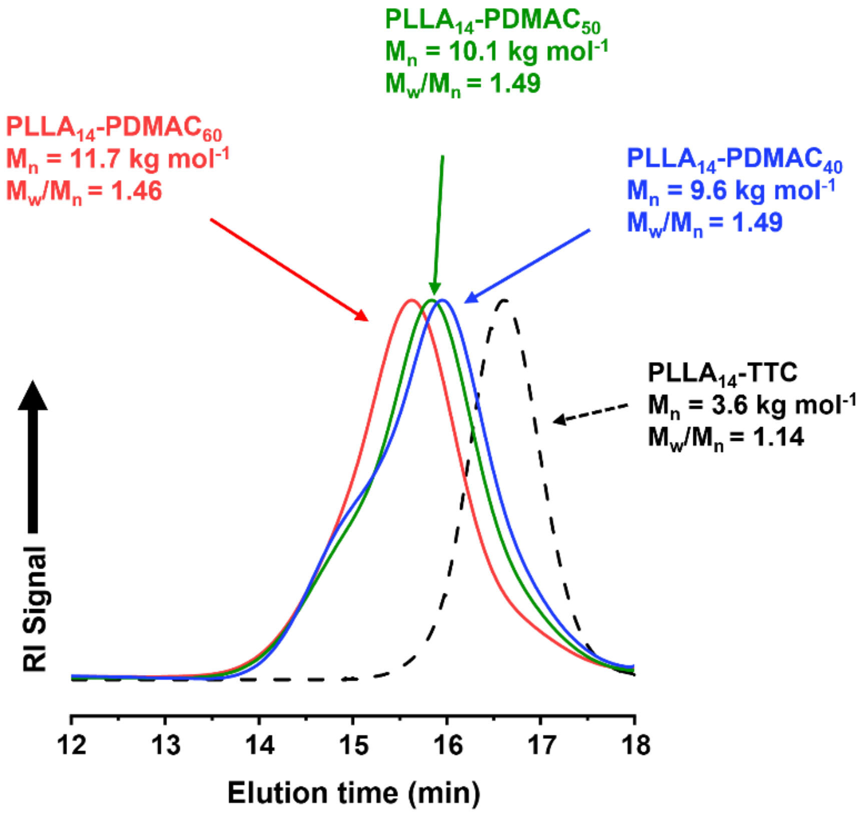

Figure S8. DMF GPC curves (refractive index detector) recorded for a series of $PLLA_{14}$-$PDMAC_{40-60}$ diblock copolymers and a $PLLA_{14}$-TTC homopolymer. Each copolymer was prepared by *reverse sequence* aqueous PISA at 90°C.

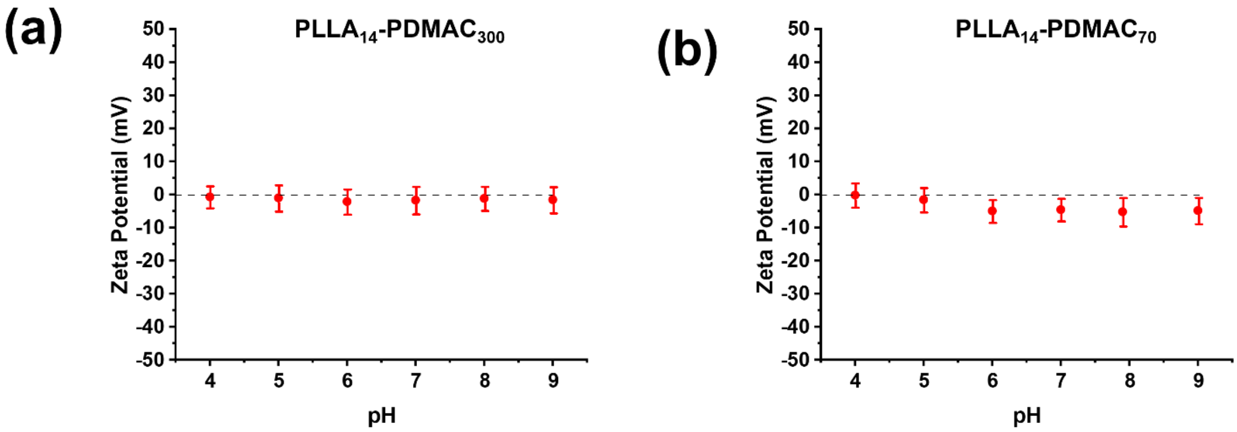

Figure S9. Zeta potential vs. pH curve recorded for a 1.0% w/w aqueous dispersion of (a) $PLLA_{14}$-$PDMAC_{300}$ nanoparticles and (b) $PLLA_{14}$-$PDMAC_{70}$ nanoparticles.

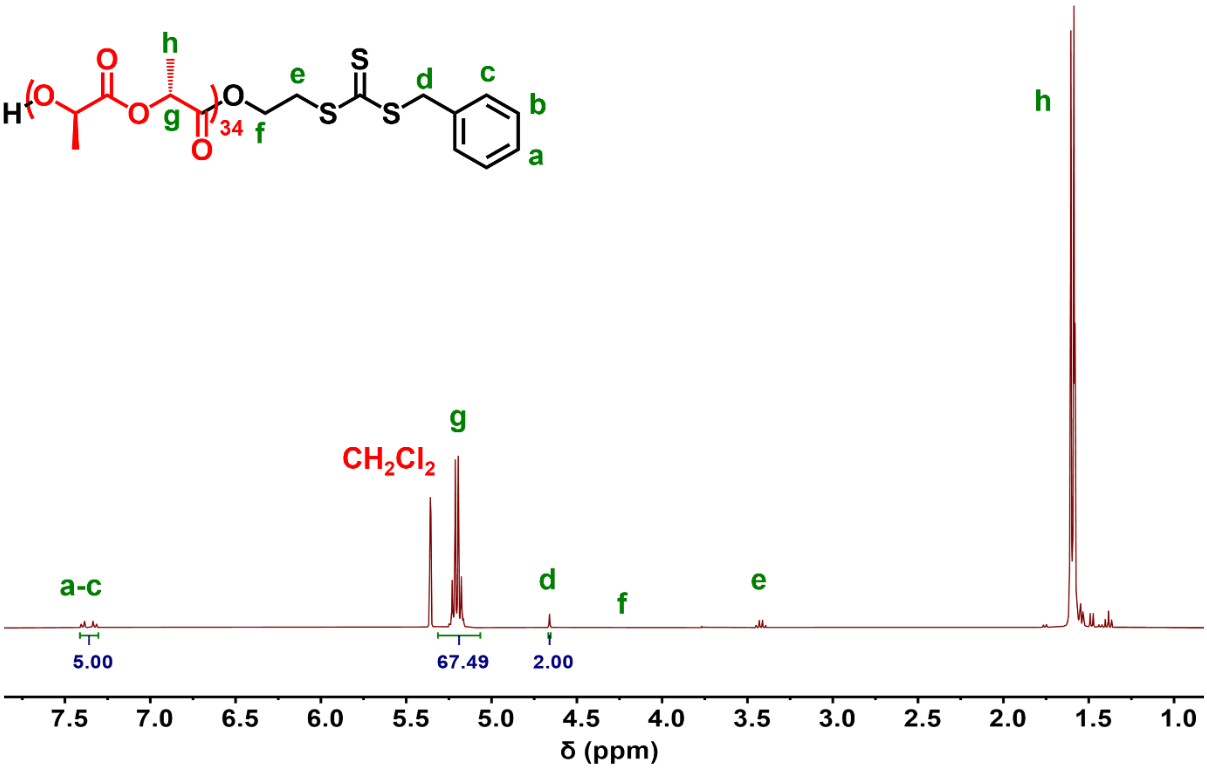

Figure S10. Assigned ^{1}H NMR spectrum (CD_2Cl_2) recorded for a $PLLA_{34}$-TTC precursor, which was prepared via DMAP-catalyzed anionic ring-opening polymerization of *L*-lactide using a hydroxy-functional RAFT agent as an initiator.

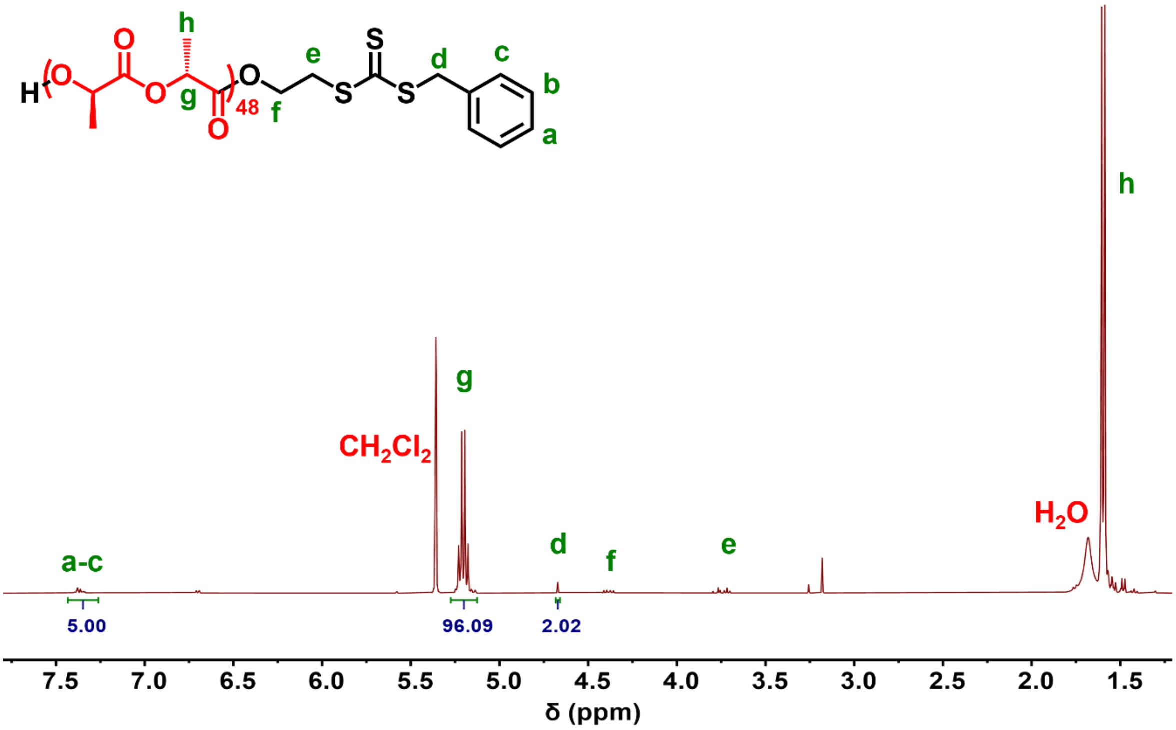

Figure S11. Assigned ^{1}H NMR spectrum (CD_2Cl_2) recorded for a $PLLA_{48}$-TTC precursor, which was prepared via DMAP-catalyzed anionic ring-opening polymerization of *L*-lactide using a hydroxy-functional RAFT agent as an initiator.

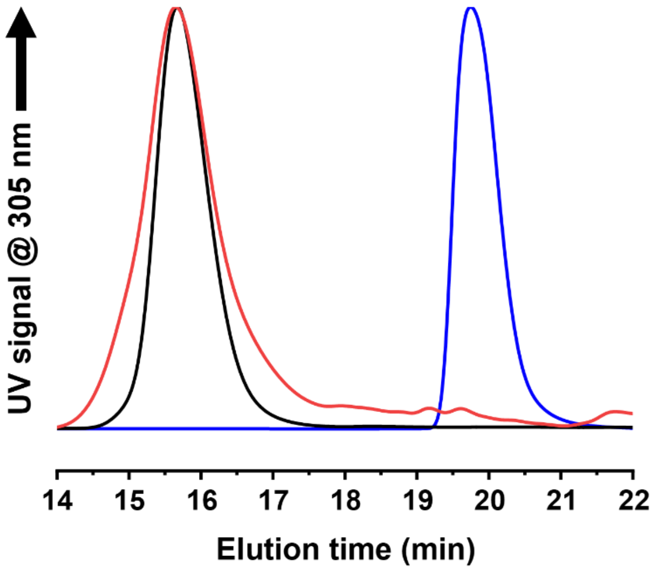

Figure S12. DMF UV GPC curves (λ = 305 nm) recorded for $PLLA_{34}$-TTC (black curve), $PLLA_{48}$-TTC (red curve) and the hydroxy-functional RAFT agent (blue curve). For GPC analysis of the RAFT agent, the DMF eluent contained 1% glacial acetic acid but no LiBr. For GPC analysis of the PLLA precursors, the DMF eluent contained 10 mM LiBr but no glacial acetic acid.

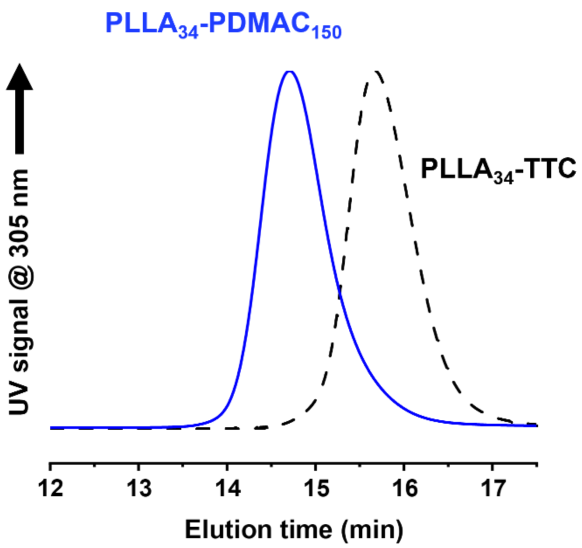

Figure S13. DMF UV GPC curves (λ = 305 nm) recorded for a $PLLA_{34}$-$PDMAC_{150}$ diblock copolymer (blue trace) and a $PLLA_{34}$-TTC homopolymer precursor (black trace). The copolymer was prepared by *reverse sequence* aqueous PISA at 70°C.

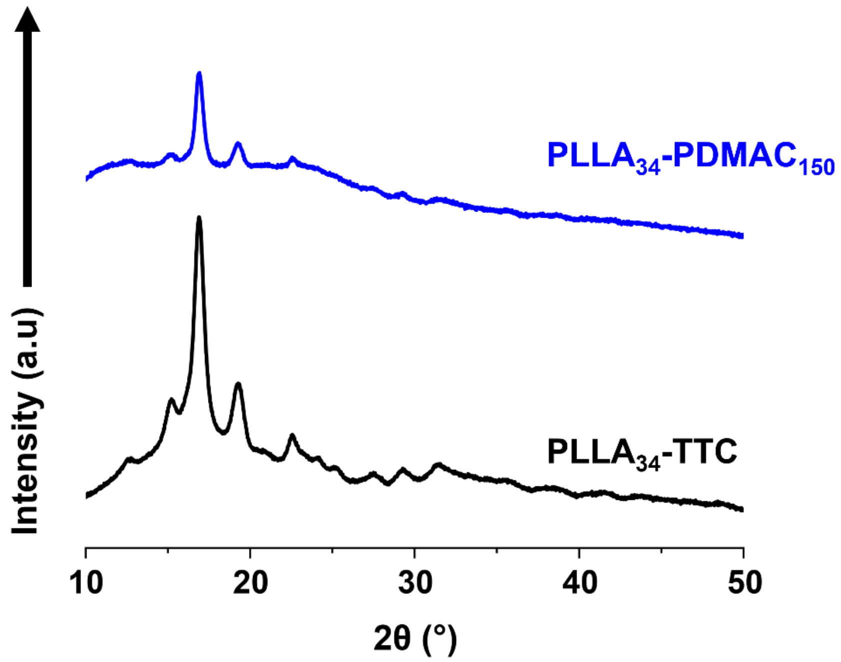

Figure S14. XRD patterns recorded for the $PLLA_{34}$-TTC precursor and freeze-dried $PLLA_{34}$-$PDMAC_{150}$ rod-like nanoparticles.

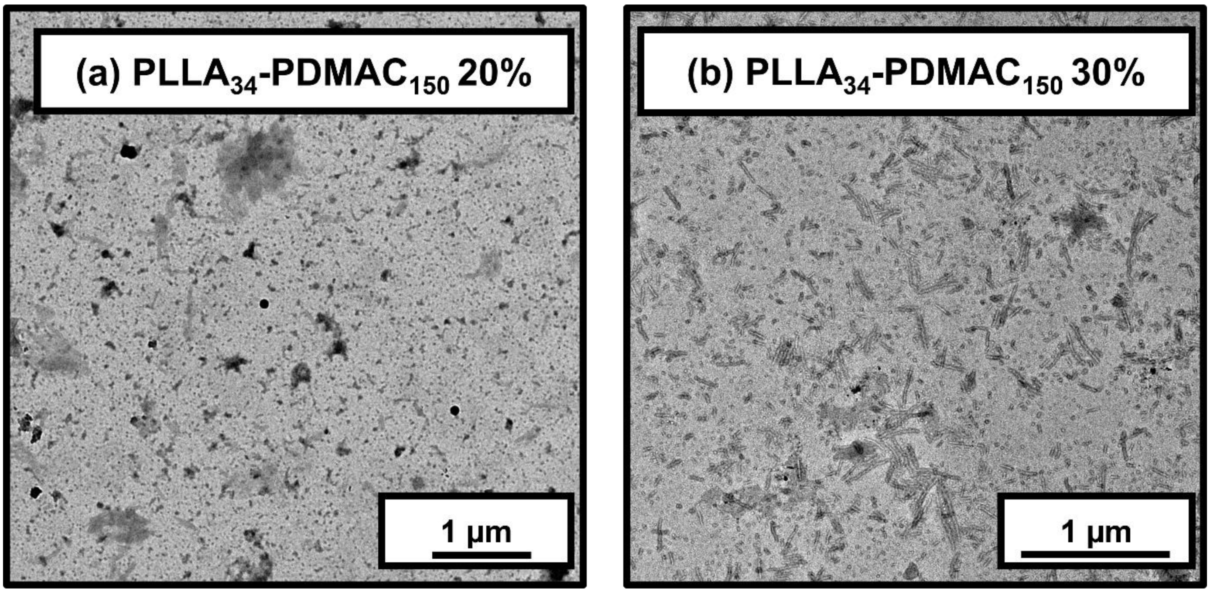

Figure S15. Representative TEM images recorded for dilute aqueous dispersions of $PLLA_{34}$-$PDMAC_{150}$ nanoparticles prepared by reverse sequence aqueous PISA at 70 ºC targeting (a) 20% w/w solids and (b) 30% w/w solids.

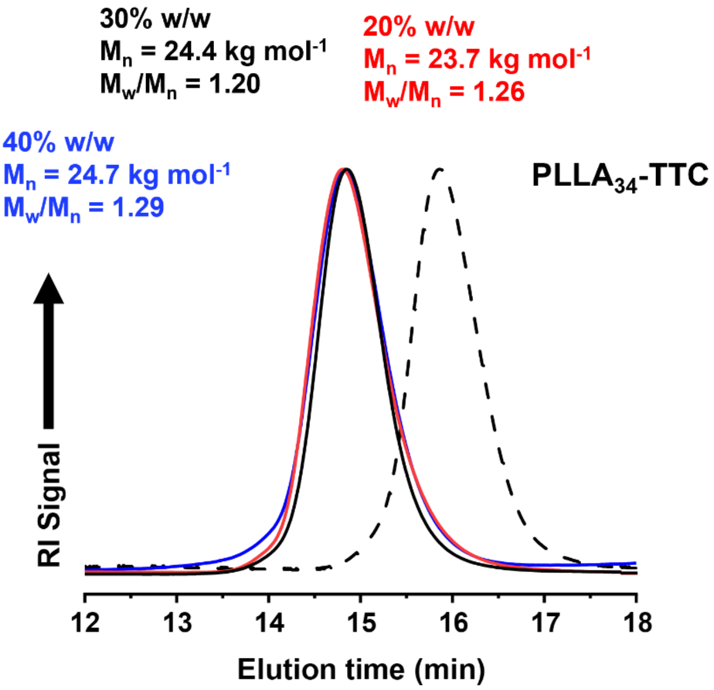

Figure S16. DMF UV GPC curves (λ = 305 nm) recorded for a series of a $PLLA_{34}$-$PDMAC_{150}$ diblock copolymer and a $PLLA_{34}$-TTC homopolymer precursor. Copolymers were prepared by *reverse sequence* aqueous PISA at 70ºC and diluted to 40% w/w (blue trace), 30% w/w (black trace) and 20% w/w (red trace) at intermediate PDMAC conversion.

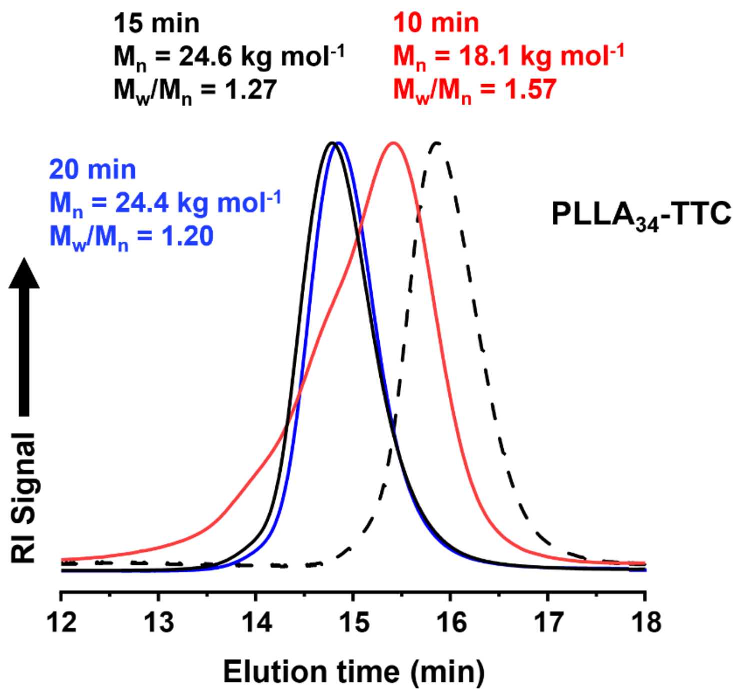

Figure S17. DMF UV GPC curves (λ = 305 nm) recorded for a series of a PLLA$_{34}$-PDMAC$_{150}$ diblock copolymer and a PLLA$_{34}$-TTC homopolymer precursor. Copolymers were prepared by *reverse sequence* aqueous PISA at 70ºC and diluted to after 10 min (red trace), 15 min (black trace) and 20 min (blue trace) of reaction time.

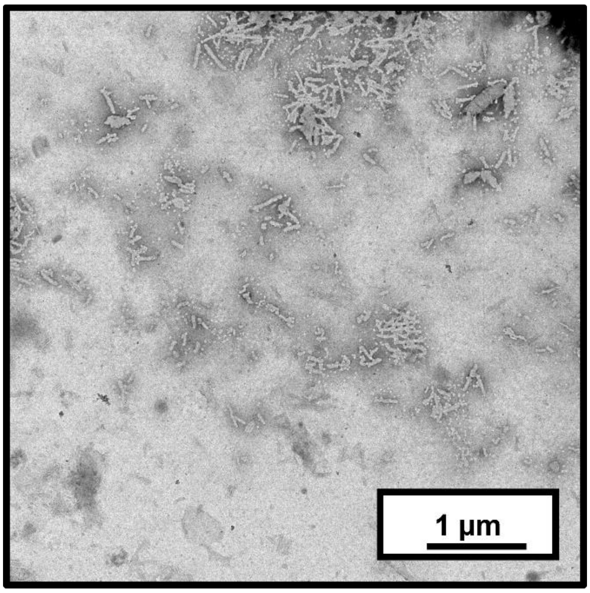

Figure S18. Representative TEM image recorded for a dilute aqueous dispersion of PLLA$_{34}$-PDMAC$_{150}$ nanoparticles prepared by reverse sequence aqueous PISA at 70 ºC by addition of degassed water after 15 min to target 30% w/w solids.

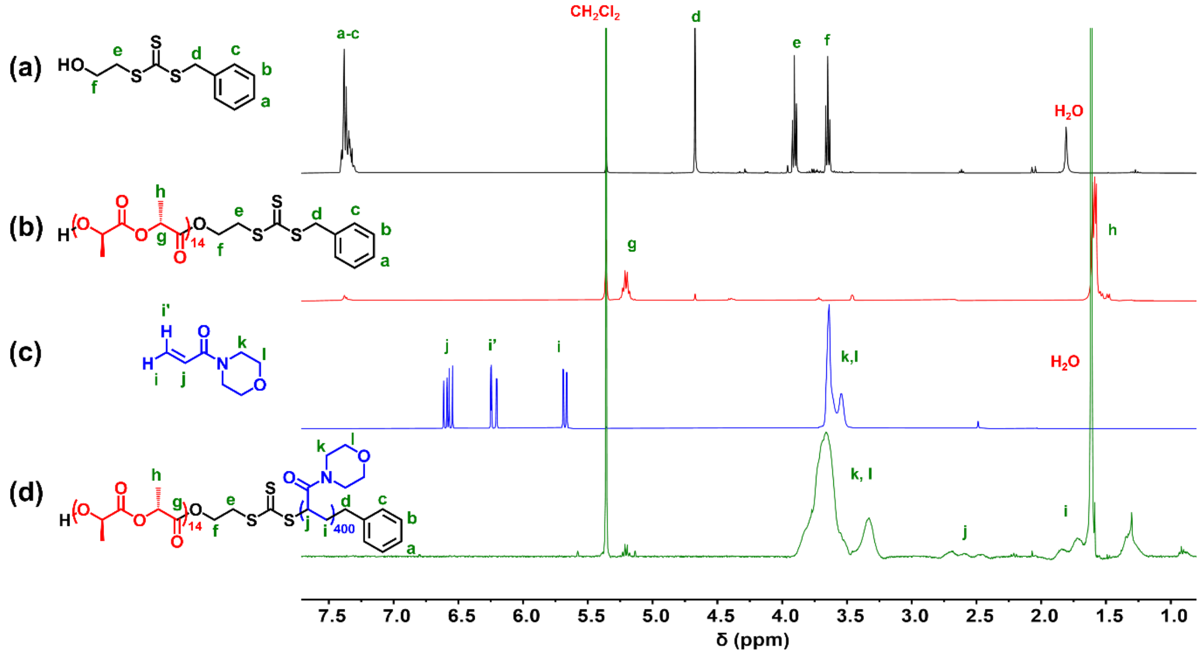

Figure S19. ^{1}H NMR spectra (CD_2Cl_2) recorded for (a) the hydroxy-functional RAFT agent, (b) the $PLLA_{14}$-TTC precursor (c) NAM monomer and (d) a $PLLA_{14}$-$PNAM_{400}$ diblock copolymer.

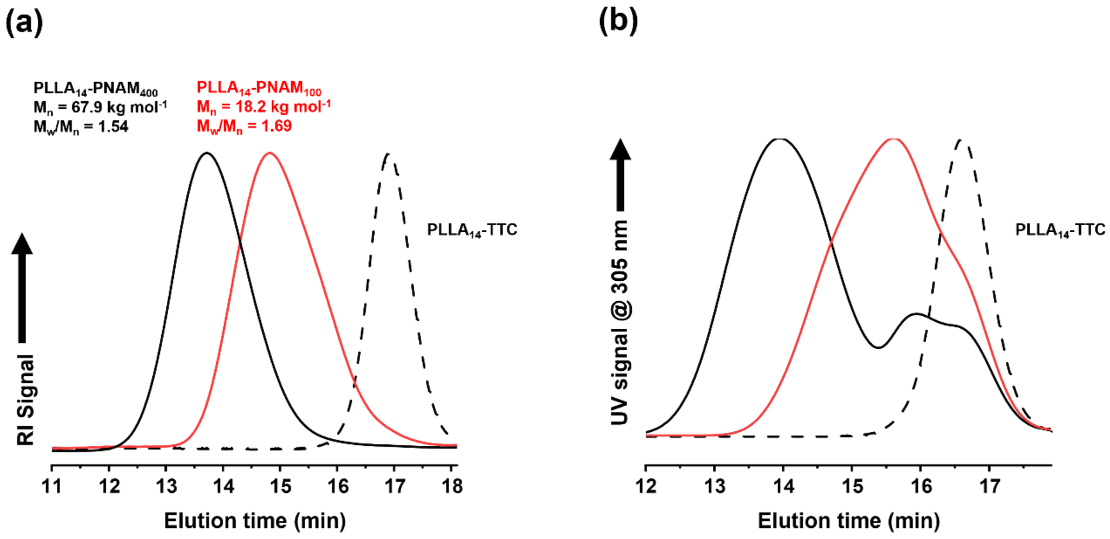

Figure S20. (a) RI GPC and (b) UV GPC curves (λ = 305 nm) recorded for a $PLLA_{14}$-$PNAM_{400}$ (black trace) and $PLLA_{14}$-$PNAM_{100}$ (red trace) diblock copolymer and a $PLLA_{14}$-TTC homopolymer precursor using a DMF eluent. These copolymers were prepared by *reverse sequence* aqueous PISA at 70ºC.

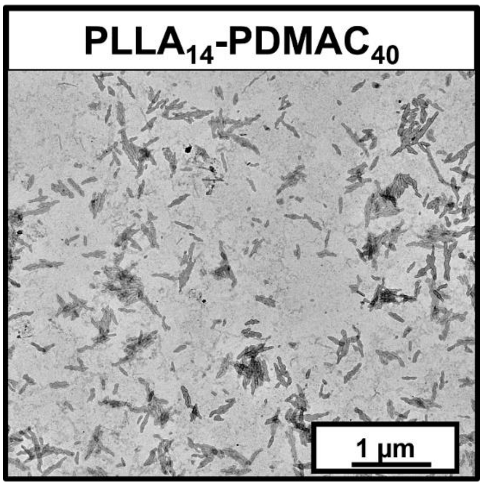

Figure S21. Representative TEM image recorded for a dilute aqueous dispersion of $PLLA_{14}$-$PDMAC_{40}$ nanoparticles prepared by reverse sequence aqueous PISA at 90 °C.

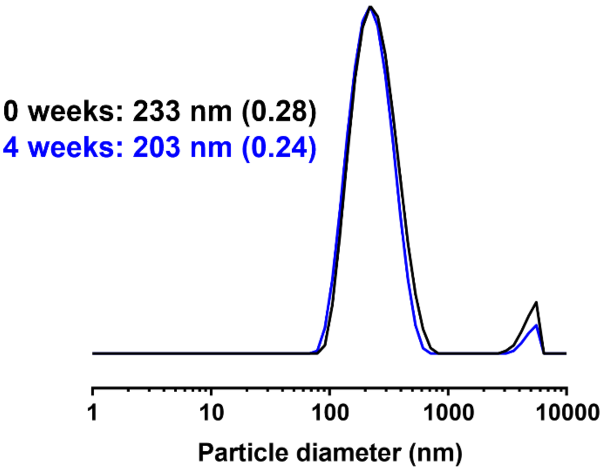

Figure S22. DLS particle size distributions recorded for freshly prepared $PLLA_{14}$-$PDMAC_{40}$ nanoparticles (black curve) and after storage for four weeks at 20°C (blue curve) as a 30% w/w aqueous dispersion in deionized water (pH 6.7). DLS measurements were made on 0.1% w/w aqueous dispersions in each case.

References

1. Skey, J; O'Reilly, R. K. Facile one-pot synthesis of a range of reversible addition-fragmentation chain transfer (RAFT) agents. *Chem. Commun.*, **2008**, 4183–4185.

2. Samarajeewa, S.; Shrestha, R.; Li, Y.; Wooley, K. L. Degradability of Poly(Lactic Acid)-Containing Nanoparticles: Enzymatic Access through a Cross-Linked Shell Barrier. *J. Am. Chem. Soc.* **2012**, *134*, 1235–1242.
